# *Ostreococcus tauri* is a new model green alga for studying iron metabolism in eukaryotic phytoplankton

**DOI:** 10.1186/s12864-016-2666-6

**Published:** 2016-05-03

**Authors:** Gaëlle Lelandais, Ivo Scheiber, Javier Paz-Yepes, Jean-Claude Lozano, Hugo Botebol, Jana Pilátová, Vojtěch Žárský, Thibaut Léger, Pierre-Louis Blaiseau, Chris Bowler, François-Yves Bouget, Jean-Michel Camadro, Robert Sutak, Emmanuel Lesuisse

**Affiliations:** CNRS, Institut Jacques Monod, Université Paris Diderot-Paris 7, F–75013 Paris, France; Department of Parasitology, Faculty of Science, Charles University in Prague, 12844 Prague, Czech Republic; Ecole Normale Supérieure, PSL Research University, Institut de Biologie de l’Ecole Normale Supérieure (IBENS), CNRS UMR 8197, INSERM U1024, 46 rue d’Ulm, F-75005 Paris, France; Sorbonne Universités, University Pierre et Marie Curie, University of Paris VI, CNRS, Laboratoire d’Océanographie Microbienne, Observatoire Océanologique, F-66650 Banyuls-sur-Mer, France

**Keywords:** Iron, Ostreococcus, Marine phytoplankton, RNA-seq analysis

## Abstract

**Background:**

Low iron bioavailability is a common feature of ocean surface water and therefore micro-algae developed original strategies to optimize iron uptake and metabolism. The marine picoeukaryotic green alga *Ostreococcus tauri* is a very good model for studying physiological and genetic aspects of the adaptation of the green algal lineage to the marine environment: it has a very compact genome, is easy to culture in laboratory conditions, and can be genetically manipulated by efficient homologous recombination. In this study, we aimed at characterizing the mechanisms of iron assimilation in *O. tauri* by combining genetics and physiological tools. Specifically, we wanted to identify and functionally characterize groups of genes displaying tightly orchestrated temporal expression patterns following the exposure of cells to iron deprivation and day/night cycles, and to highlight unique features of iron metabolism in *O. tauri*, as compared to the freshwater model alga *Chalamydomonas reinhardtii*.

**Results:**

We used RNA sequencing to investigated the transcriptional responses to iron limitation in *O. tauri* and found that most of the genes involved in iron uptake and metabolism in *O. tauri* are regulated by day/night cycles, regardless of iron status. *O. tauri* lacks the classical components of a reductive iron uptake system, and has no obvious iron regulon. Iron uptake appears to be copper-independent, but is regulated by zinc. Conversely, iron deprivation resulted in the transcriptional activation of numerous genes encoding zinc-containing regulation factors. Iron uptake is likely mediated by a ZIP-family protein (Ot-Irt1) and by a new Fea1-related protein (Ot-Fea1) containing duplicated Fea1 domains. The adaptation of cells to iron limitation involved an iron-sparing response tightly coordinated with diurnal cycles to optimize cell functions and synchronize these functions with the day/night redistribution of iron orchestrated by ferritin, and a stress response based on the induction of thioredoxin-like proteins, of peroxiredoxin and of tesmin-like methallothionein rather than ascorbate. We briefly surveyed the metabolic remodeling resulting from iron deprivation.

**Conclusions:**

The mechanisms of iron uptake and utilization by *O. tauri* differ fundamentally from those described in *C. reinhardtii*. We propose this species as a new model for investigation of iron metabolism in marine microalgae.

**Electronic supplementary material:**

The online version of this article (doi:10.1186/s12864-016-2666-6) contains supplementary material, which is available to authorized users.

## Background

Low iron bioavailability is a common feature of ocean surface water and alkaline soils, and the mechanisms of iron uptake and metabolism have been thoroughly studied in photosynthetic organisms. The unicellular alga *Chlamydomonas reinhardtii* is an excellent model for investigations of the crucial mechanisms involved in the uptake of iron and other important metals, and in the physiological response to iron deficiency in unicellular photosynthetic organisms [46]. The main iron uptake system of *C. reinhardtii* is a reductive system, very similar to that described in yeast: a plasma membrane reductase (Fre) mediates the reductive dissociation of extracellular ferric complexes, and iron is taken up by channeling through a permease (Ftr) associated with a multicopper oxidase (Fox) that re-oxidizes iron during its uptake (reviewed in [24]). The best-studied model land plant is *Arabidopsis thaliana*, which also uses a reductive system (“strategy 1”, which also involves the excretion of phenolics and protons to solubilize iron), but takes up iron as ferrous ions without concomitant copper-dependent re-oxidation, via ZIP family proteins (Irt) (reviewed in [13]). Finally, graminaceous plants can take up iron non-reductively (strategy 2), through the excretion of phytosiderophores (reviewed by [34].

Reductive iron uptake probably also occurs in marine microalgae (reviewed by [48]), but other high-affinity mechanisms for iron uptake likely also occur in these marine organisms, several species of which have no ferrireductase activity [68]. Evidence is emerging for the existence of siderophore-independent, nonreductive iron uptake systems able to take up hydrated ferric species without prior reduction. A protein widely expressed by marine phytoplankton (Isip2a) was recently shown to participate in such a system, by concentrating ferric iron at the cell surface [49]. However, the molecular mechanisms involved in iron uptake by marine microalgae and, more generally, the precise mechanisms of cell adaptation to iron scarcity in the marine environment remain very poorly understood, particularly for the green algal lineage.

The cosmopolitan marine microalga *Ostreococcus tauri* is an ancient member of the green algal lineage (Prasinophyceae). Several of its features make it a very good model for studying physiological and genetic aspects of the adaptation of the green algal lineage to the marine environment. This microalga is the smallest eukaryotic organism described to date, it has a very compact genome [20], is easy to culture in laboratory conditions, and can be genetically manipulated by efficient homologous recombination [44]. We have shown that this species has no inducible ferrireductase activity at the cell surface [68], and that it uses ferritin to recycle intracellular iron as a function of the day/night cycles [10]. Ferritin (FTN) also seems to be involved in iron uptake in this species, because a ∆*Ftn* mutant was found to have impaired iron uptake [10]. Surprisingly, an analysis of the genomes of *O. tauri* and *O. lucimarinus* provided no clues to the iron-uptake mechanisms used by these species [52].

We investigated iron homeostasis in *O. tauri* by evaluating the short-term and long-term adaptive responses of this species to iron deprivation by RNA sequencing (RNAseq) in combination with physiological assays. We paid particular attention to the transcriptional response of cells to iron deprivation according to the day/night cycles, as previous studies had reported an orchestration of the transcription of biological processes around these cycles in *O. tauri* [47]. Our aim in this work was to identify clusters of genes displaying tightly orchestrated temporal expression patterns following the exposure of cells to iron deprivation and day/night cycles. We also aimed to compare the cellular response to iron deprivation in two green algae, *O. tauri* and *C. reinhardtii*, based on the extensive work of Urzica *et al.* on *C. reinhardtii* [72]. We found that the genes involved in iron uptake and metabolism were mostly regulated according to the day/night cycles [10]. We further found key differences in iron metabolism between *O. tauri* and *C. reinhardtii*, leading us to conclude that these two species do not use the same iron uptake system and do not respond in the same manner to changes in iron nutritional status. The transcriptional response of *O. tauri* to iron deprivation was unique within the green alga lineage. We therefore propose the use of this species as a new model for studies of iron metabolism in eukaryotic phytoplankton.

## Results

### Global transcriptomic analysis reveals that iron homeostasis is tightly coordinated by day/night cycles

**Fig. 1:**
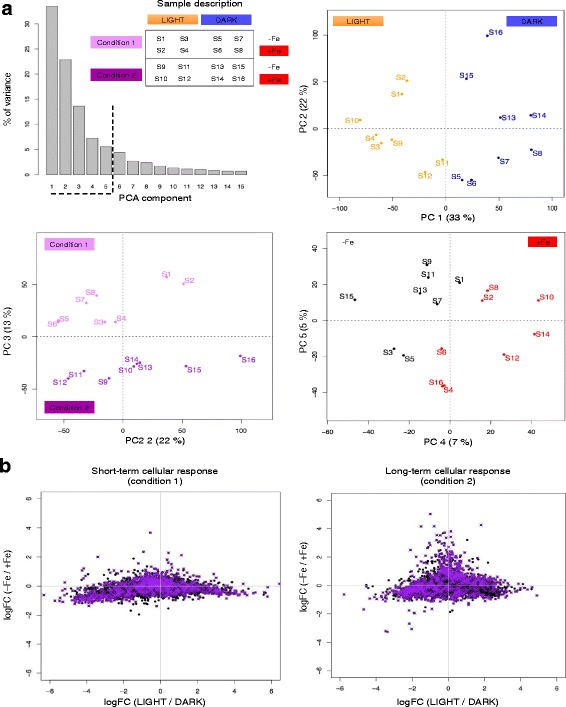
Global analysis of changes in mRNA abundance. Gene expression data for all genes and all samples were analyzed by PCA. PCA is a well-established technique in multivariate statistics. The general principle is to determine new coordinate systems such that the first coordinate explains the maximal amount of variance in the data and successive coordinates explain maximal remaining variance whilst being orthogonal to the first [16]. **a** Barplot showing the variability accounted for by each PCA component. Components 1 and 2 accounted for 33 and 22 % of the global variance, respectively. Components 3, 4 and 5 accounted for 13, 7 and 5 % of the global variance. Biplots representing the RNAseq samples on the coordinate systems defined by PCA components 1 and 2 (*upper right*), PCA components 2 and 3 (*lower left*) and PCA components 4 and 5 (*lower right*). **b** Plots of LogFC values calculated with gene expression measurements for LIGHT and DARK samples (*x*-axis) or for –Fe and + Fe samples (*y*-axis). Points represent genes. Positive LogFC values on the *x*-axis indicate higher levels of gene expression in the *light* than in the *dark*,, whereas positive LogFC values on the *y*-axis indicate higher levels of gene expression –Fe than in + Fe conditions. The 1049 genes selected for further characterization are shown in purple (see the main text and Additional file 4: Data Sets 1)

We used RNAseq to obtain a genome-wide view of the cellular response of *O. tauri* to iron deprivation. As we aimed to investigate the fundamental aspects of iron metabolism in *O. tauri*, we defined two different sets of experiments (Additional file 1: Figure S1; in both sets of experiments, the cells were grown under a 12 h:12 h light/dark regime). In one set of experiments (“condition 1”), we analyzed the transcriptome of the cells shortly (3 h and 6 h) after shifting the cells to iron-rich and iron-deficient conditions during the day and during the night (see Methods). These experiments were designed to characterize the short-term response of cells subjected to sudden and massive changes in iron availability. In a second set of experiments (“condition 2”), we carried out a series of transcriptome analyses over a 24-h period (3 h, 9 h, 15 h and 22 h after dawn) after the cells had been allowed to adapt to iron-poor or iron-rich conditions for one week (see Methods). These experiments were designed to characterize the long-term adaptive response of cells to changes in iron concentration. All experiments were conducted in triplicate and we applied a dedicated bioinformatics framework for analysis of the RNAseq results (Additional file 2: Figure S2). In total, we sequenced, analyzed and combined the results from 48 mRNA samples from algae grown in 16 different sets of growth conditions (S_1_ to S_16_, Additional file 1: Figure S1 and Fig. 1). This is, to our knowledge, the largest time-course transcript profiling study ever performed in *O. tauri*.

Our RNAseq samples describe the transcriptome states of *O. tauri* associated with three different factors: *i*) iron deprivation or excess (referred to as “-Fe” and “+Fe” conditions), *ii*) day or night (referred to as light and dark), and *iii*) short- or long-term adaptive responses of the cells (referred to as condition 1 and condition 2) (Fig. 1a and Additional file 1: Figure S1). We first quantified the influence of these factors on the global variability observed in our RNAseq results. We carried out principal component analysis (PCA) on the complete gene expression dataset (all genes and all samples analyzed simultaneously). We found that the percentage of the variance decreased rapidly with increasing principal component (PC) number, with more than 95 % of the total variance accounted for by only five PCs (Fig. 1a). This analysis identified the key factors affecting gene expression. In particular, PC1 and PC2, which accounted for 55 % of the variance, define a coordinate system in which samples are clearly grouped according to light/dark, whereas PC2 and PC3 (35 % of the variability) define a coordinate system in which samples are grouped according to experimental conditions (1 or 2) (Fig. 1a). The iron nutritional status also had an impact on gene expression (PC4 and PC5, accounting for 12 % of the variability) but of a lesser magnitude than that of the other factors. The expression of genes regulated by iron nutrition is thus strongly affected by day/night cycles, particularly in condition 2, in which cells were allowed to adapt to low/high levels of iron over several day/night cycles. It has been shown that the transcription of genes involved in biological processes in *O. tauri* is organized principally around day/night cycles under standard growth conditions [19, 47]. We show here that the light/dark factor retains its predominant position under conditions of iron stress, suggesting that iron homeostasis is regulated according to the day/night conditions.

### Transcriptional regulation in response to iron deprivation is a robust cellular process that can be maintained for extended periods

Among the genes for which expression data were available, we identified those displaying significantly different levels of expression in conditions of iron deprivation (−Fe) and iron excess (+Fe). We used the DEseq program [3] to identify differentially expressed genes and to calculate a risk of error (*p*-value). The numbers of genes with a *p*-value < 1 % and a LogFC > 1 (for upregulated genes) or LogFC < −1 (for downregulated genes) in each condition are shown in Additional file 3: Figure S3A. We found that 224 and 259 genes were downregulated in condition 1 and condition 2, respectively, and that this downregulation was mostly specific to the experimental conditions used, consistent with the PCA results (see above). However, a different situation was observed for upregulated genes. We found that 1,201 genes were upregulated after a prolonged period of cell adaptation to iron deficiency (condition 2), whereas only 128 genes were upregulated after short-term iron stress (condition 1), with most of the genes (75 %) upregulated in condition 1 also being upregulated in condition 2. Thus, genes induced less than 6 h after the shift to iron-deficient conditions were generally still induced after one week of adaptation to iron shortage (Additional file 3: Figure S3A), highlighting the robustness of the cellular response to iron stress.

### Iron metabolism in O. tauri involves many new proteins and pathways that remain to be discovered

**Fig. 2:**
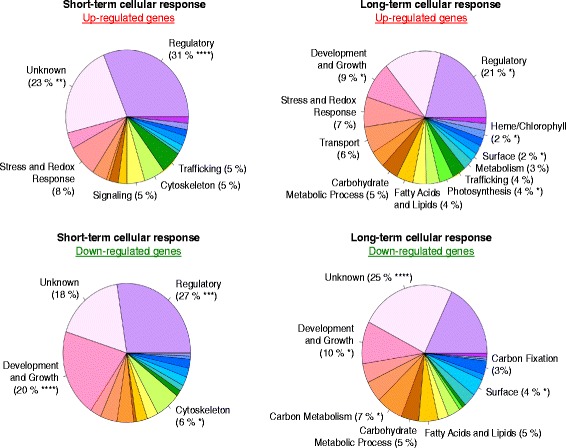
Functional categories associated with the genes up- and downregulated in response to iron deprivation. Pie charts representing the 19 functional categories defined in this work (see Additional file 3: Fig. S3 for a detailed description), with the percentage of genes assigned to each function. Only the functions for which the calculated enrichment *p*-value < 0.15 are noted. (****) indicates a *p*-value < 0.0001, (***) indicates a *p*-value < 0.001, (**) indicates a *p*-value < 0.01 and (*) indicates a *p*-value < 0.05

We defined a set of genes including *i*) genes differentially expressed in a least two conditions, and *ii*) genes differentially expressed in only one condition but for which interesting functions were retrieved (Additional file 3: Figure S3B). For that, we developed a dedicated process for inferring gene function, combining information from multiple web resources (ORCAE, HHPRED and PFAM databases) and systematic manual inspection of the retrieve predictions (see Methods). We then defined 19 general functional categories (and associated subfunctions) and used them to classify 1,048 genes (Additional file 3: Figure S3B). This list of genes, together with detailed information including function, subfunction and database search results, is presented in Additional file 4: Data Sets 1. The data corresponding to all *O. tauri* genes are presented in Additional file 5: Data Sets 2. Functions associated with the up- and downregulated genes are shown in Fig. 2. In condition 1, about two thirds of the upregulated genes belonged to the “Regulatory” (31 %), “Unknown” (23 %) or “Stress and redox response” (8 %) functional groups. The *p*-values associated with the functional enrichment of genes were calculated (see Methods) and the result was highly significant for the “Regulatory” function (*p*-value < 10^−4^) and particularly for the “Zinc-containing proteins” subfunction (see Additional file 4: Data Sets 1). The genes rapidly downregulated by iron deprivation were particularly remarkable in terms of their highly significant enrichment in the function “Development and growth” (*p*-value < 10^−4^), including the “Ribosome-related” subfunction (see Additional file 4: Data Sets 1). These data suggest that the early response of cells to iron deprivation involves the recruitment of a large number of regulatory proteins (many of which are zinc-containing proteins) and a decrease in protein synthesis through repression of the translational machinery. This decrease in translation is consistent with the observation that iron limitation decreases the total amount of protein per cell as well as cell size [42]. The massive recruitment of zinc-containing proteins (56 genes upregulated by iron deprivation encode zinc-containing proteins, mostly zinc finger proteins; Additional file 4: Data Sets 1) raises questions about the link between zinc and iron in *O. tauri* (see below). Condition 2 led to the differential expression of many more genes and the range of functional categories for upregulated genes was broader than for condition 1 (Fig. 2). In addition to the functional enrichments already observed in the early response (see above), functional enrichments (*p*-values < 0.15) were observed for the functions “Metabolism” (3 %), “Photosynthesis” (4 %), “Surface” (2 %) and “Heme/Chlorophyll” (2 %). Most of the genes downregulated by iron deprivation were associated with the functions “Development and Growth” (10 %), “Carbon Metabolism” (7 %), and “Surface” (4 %). These data are consistent with the metabolic remodeling expected during the adaptation of cells to iron limitation. Surprisingly, 25 % of the downregulated genes were assigned to the functional category “Unknown”, a percentage much higher than would be expected by chance alone (enrichment *p*-value < 10^−4^). As proposed by Urzica *et al.* [72], these proteins can be considered “pioneer proteins”, because they contain no domains suggestive of a particular function. Overall, such pioneer proteins accounted for 18 % of the genes displaying up- or downregulation by iron. This very high value strongly suggests that iron metabolism in *O. tauri* involves more currently unknown proteins and pathways than that in other model organisms of the green plant lineage, such as *C. reinhardtii* [8, 24] and *A. thaliana* [13, 36].

### The green algae O. tauri and C. reinhardtii display fundamental differences in iron metabolism

In a previous study, Urzica *et al.* [72] surveyed the iron nutrition-responsive transcriptome of *C. reinhardtii* with RNAseq technology. We carried out a genome-wide comparison of the transcriptional programs involved in iron metabolism in *O. tauri* and *C. reinhardtii* by collecting the RNAseq data from the work of Urzica *et al.* [72] and inferring orthology relationships between all coding sequences of *O. tauri* and *C. reinhardtii* (with the INPARANOID algorithm [60]). Surprisingly, we found that many proteins of functional importance in the metabolism of iron by *C. reinhardtii* had no orthologs in *O. tauri*, or that the orthologs of these proteins in *O. tauri* were not affected by iron nutrition in our dataset (Additional file 6: Figure S4). This was the case, for example, for the target genes studied by Urzica *et al.* [72] *VTC2* and *MDAR1* (involved in ascorbate synthesis), *CDG27*, and the homolog of the BRUTUS gene of *A. thaliana* (Cre05.g248550). These genes encode proteins thought to play important roles in iron metabolism in green plants, but their homologs in *O. tauri* (ostta04g05300, ostta05g00840, ostta18g01260 and ostta11g00420, respectively) are not regulated by iron (Additional file 5: Data Sets 2), although we cannot exclude the possibility that these genes could be regulated at the post-transcriptional level in *O. tauri*. These fundamental differences between the two green algae were confirmed by measurements of the growth rates of the two species in media containing a gradient of iron and copper concentrations (Additional file 7: Figure S5). *O. tauri* cells reached maximal growth rates at iron concentrations two orders of magnitude lower than those for *C. reinhardtii*, and copper supplementation improved the growth of *C. reinhardtii,* but not that of *O. tauri* cells (Additional file 7: Figure S5).

**Fig. 3:**
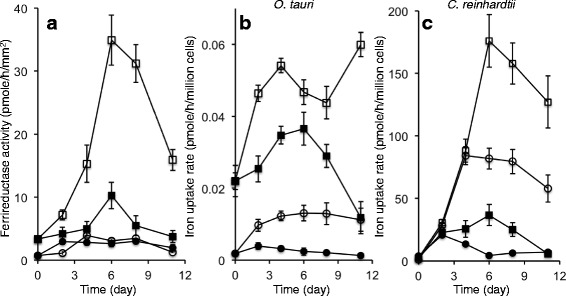
*Ferrireductase* activity and iron uptake activities of *O. tauri and C. reinhardtii. O. tauri* and *C. reinhardtii* cells were grown for one week in their respective standard media (see Methods), washed once in iron-free medium and they were then used to inoculate Mf (*O. tauri*) or modified TAP medium (*C. reinhardtii*) with either no added iron (open symbols) or with the addition of 5 μM Fe(III)-citrate (closed symbols). Cells were harvested at intervals, and ferrireductase activity (A) and iron uptake activities (B, C) were determined. **a** Ferrireductase activity was measured as described in the methods. Circles: *O. tauri*; squares: *C. reinhardtii*. Values are expressed in pmole/h/mm^2^ of cell surface to facilitate comparison (see in Methods). **b** Iron uptake by *O. tauri* cells, measured with either 1 μM Fe(II)-ascorbate (1:1000) (*squares*) or 1 μM Fe(III)-EDTA (1:5) (*circles*). **c** Iron uptake by *C. reinhardtii* cells, measured with either 1 μM Fe(II)-ascorbate (1:1000) (*squares*) or 1 μM Fe(III)-EDTA (1:5) (*circles*). Means ± SD from 3 experiments

Like most organisms, *O. tauri* and *C. reinhardtii* regulate their iron uptake systems as a function of iron availability, but we found that they did not regulate their uptake systems for ferric and/or ferrous iron in the same way following a rapid decrease in the iron concentration of the medium (Fig. 3). *O. tauri* had very low levels of ferrireductase activity (about 0.01 nmole/h/million cells), as previously reported [68], that were not induced by iron deprivation in any condition (Fig. 3a). In *C. reinhardtii*, the induction of ferrireductase activity was maximal about five to six days after the shift to iron-deficient conditions (reaching about 15 nmole/h/million cells; Fig. 3a). Unlike ferrireductase activity, iron uptake (from both ferric and ferrous iron) by *O. tauri* was induced shortly after the cells were shifted to iron-deficient conditions (Fig. 3b), and ferrous, but not ferric iron uptake was further induced subsequently, after more than one week of iron deprivation (Fig. 3b). In *C. reinhardtii,* both ferrous and ferric iron uptake activities were maximally induced, together with ferrireductase activity [2], after a few days of growth in iron-deficient conditions (Fig. 3c). The observation that *O. tauri* cells can induce iron uptake from a ferric iron source (ferric EDTA) without the parallel induction of a ferrireductase system suggests that this organism, unlike *C. reinhardtii*, can take up iron without prior reduction, as recently demonstrated for the diatom *P. triconutum* [49].

### Iron metabolism in green algae: clues from comparative transcriptomics in O. tauri and C. reinhardtii

#### O. tauri lacks the classical components of a reductive iron uptake system

In *C. reinhardtii*, the inducible ferrireductase FRE1 is involved in iron uptake [2, 7], and another putative ferrireductase (Cre05.g241400) induced by iron starvation is thought to function in intracellular iron metabolism [72]. The *O. tauri* ferrireductase homolog (ostta09g01890) was strongly expressed (mean read number per kb (RNK) of 3,676) but was slightly repressed by iron deprivation, and would therefore not be expected to play a major role in iron uptake (Additional file 5: Data Sets 2). However, two cytochrome b561 homologs (ostta16g00370 and ostta04g02840) were significantly upregulated by iron deprivation (Table 1), although their levels of expression were low (RNKs of 103 and 146, respectively), and these two genes were induced only after prolonged growth in iron-deficient conditions (condition 2) incompatible with the rapid upregulation of ferrireductase activity in organisms using a reductive uptake system [2, 40, 73]. However, cytochrome b561 is known to have ferrireductase activity [6, 67, 70] and we cannot rule out the possibility that these genes participate in iron uptake in some way. In *C. reinhardtii*, two cytochrome b561 family proteins containing a DOMON domain are thought to function as ferrireductases: TEF22 may be required for reductive iron use by the mitochondria, whereas Cre14.g609900 may be located at the plasma membrane [72]. The *O. tauri* cytochrome b561 homolog ostta04g02840 has a similar domain organization and is predicted to be mitochondrial (Additional file 8: Figure S6). The second cytochrome b561 homolog (ostta16g00370) has no DOMON domain and its location within the cell is unknown. This protein displays greater sequence similarity to a different cytochrome b-561-like protein (Cre06.g280100) of *C. reinhardtii* that does not appear to be regulated by iron [72] (Additional file 8: Figure S6). As no inducible ferrireductase activity was found in *O. tauri* cells (Fig. 3a), cytochrome b561 proteins may be involved in intracellular iron use rather than in iron uptake.

**Table 1:** Effect of short-term (condition 1) and long-term (condition 2) iron deprivation on abundance of RNAs encoding proteins of various functions

General Function	ORF	Protein	Condition 1			Condition 2		
			Day	Night	Mean	Day	Night	Mean
Fatty acids and lipids	ostta13g01040	∆6 fatty acid desaturase	-	-	-	++	0	+
	ostta05g00100	fatty acid desaturase	-	0	-	++	0	-
	ostta11g01160	phosphatidyl serine synthase	+	0	+	+	++	+
	ostta20g00030	malonyl-CoA:ACP transacylase	++	+	+	++	++	++
	ostta01g03840	phospholipase D	+	+	+	++	+	+
	ostta07g03850	serine incorporator	0	0	0	++	++	++
	ostta10g01650	heme oxygenase	0	0	0	+	+	+
Heme/chlorophyll	ostta12g01390	pheophorbide *a* oxygenase	-	-	-	-	+	-
	ostta01g02150	pheophorbidase	-	+	-	++	++	+
	ostta07g02450	chlorophyllide *a* oxygenase	-	0	-	0	+	-
	ostta02g03400	ferritin	+	0	0	0	+	0
Iron uptake and homeostasis	ostta18g01980	OtFea1	++	++	++	++	++	++
	ostta16g02300	ZIP family transporter	+	+	+	++	+	+
	ostta06g02310	ZIP family transporter, OtIrt1	INF	+	+	++	++	++
	ostta04g02840	cytochrome b561 homolog	0	-	0	++	++	+
	ostta16g00370	cytochrome b561 homolog	+	0	0	+	++	+
	ostta14g02780	heavy metal efflux pump	+	0	0	++	++	++
Photosynthesis	ostta12g00550	subunit B of the cytochrome *b*_*6*_ *f* complex, PetN	0	+	0	+	++	+
	ostta12g00420	PSII reaction center PsbM	0	+	0	+	++	+
	ostta10g02860	phytoene dehydrogenase or desaturase	-	0	-	0	-	-
Regulatory	ostta14g01760	RWP-RK transcription factor	++	++	++	++	++	++
	ostta03g01100	zinc GATA factor	+	+	+	+	+	+
	ostta07g03410	zinc GATA factor	++	+	+	++	+	+
	ostta10g01420	thioredoxin/glutaredoxin	+	+	+	0	++	+
Stress and redox response	ostta16g00515	GIY-YIG nuclease	0	0	0	++	++	++
	ostta01g02840	glutathione-S-transferase	+	+	+	0	++	+
	ostta18g00200	pyridine nucleotide-disulphide oxidoreductase	+	++	+	+	+	+
	ostta16g00420	ferredoxin-thioredoxin reductase	+	++	+	+	+	0
	ostta09g04220	tesmin-like methallothionein	+	+	+	+	++	+
	ostta10g01310	thioredoxin/glutaredoxin	+	+	+	0	++	+
	ostta07g03420	CCR4-Not complex	++	+	+	0	+	+
	ostta08g01350	zinc chaperone, DNAJ domain.	+	++	+	++	+	+
	ostta01g00980	vacuolar-type H^+^-pyrophosphatase	+	+	-	+	+	0
Transport	ostta06g04110	nucleobase cation symporter-1	+	+	+	++	++	++
	ostta02g02460	inorganic phosphate transporter	-	-	-	0	--	--
	ostta10g00950	nitrate transport	-	-	-	--	-	-

For each condition, we calculated the mean values of logFC (−Fe/+Fe) for the two time-points in the light (day), the two time-points in the dark (night), and the four time-points of the experiments (mean). Values are symbolized by the signs + (logFC > 0.2 < 1), ++ (logFC > 1), − (logFC < −0.2 > −1) and – (logFC < −1). Precise values for each time-point are given in Additional file 4: Data sets 1 and Additional file 5: Data sets 2

#### Zinc seems to play an important role in regulating iron uptake, but there is no clear iron-copper connection in O. tauri

The copper-dependent re-oxidation of iron during its uptake is a key feature of the iron-copper connection in eukaryotic cells [31]. However, the copper dependence of iron uptake in *O. tauri* is less marked than that in yeast and *C. reinhardtii*. There was no growth defect in the absence of added copper (Additional file 7: Figure S5), and the iron uptake rates of cells grown in copper-free Mf medium and cells grown in Mf medium supplemented with copper (10–100 nM) were not significantly different (Additional file 9: Figure S7). The putative copper transporter ostta04g03950 (homologous to the *A. thaliana COPT2* gene product) was unaffected by iron limitation (Additional file 5: Data Sets 2). This suggests that iron and copper metabolisms are not tightly connected in *O. tauri*. The closest *O. tauri* homolog to the *C. reinhardtii* multicopper oxidase Fox1 (ostta14g01670) has a different domain organization (Additional file 10: Figure S8A). This gene was only weakly expressed (113 RNK) and was not significantly induced by iron deprivation (Additional file 5: Data Sets 2). Finally, there is no clear homolog of Ftr1 in *O. tauri* (Additional file 10: Figure S8B). The classical components involved in the inducible reductive iron uptake system (reductase/multicopper ferrioxidase/permease) are therefore lacking in *O. tauri,* as previously reported [52]. Interestingly, as suggested above, zinc seems to play a more important role than copper in iron uptake in *O. tauri*. We depleted *O. tauri* cells of zinc, by growing them in Mf medium with no added zinc for 3 months and compared iron uptake kinetics between zinc-replete and zinc-deprived cells. Zinc-deprived cells displayed a deregulation of iron uptake, with higher rates of iron uptake than in control cells when the cells were grown in iron-rich medium (which suggests that zinc is required to repress iron uptake by the cells when iron concentration is high), and lower iron uptake rates than control cells when the cells were grown in iron-depleted medium (which suggests that zinc is required for the full induction of iron uptake by the cells at low iron concentration) (Additional file 9: Figure S7). Consistent with our transcriptomic data (Additional file 4: Data Sets 1), this result suggests that zinc plays a more important role in the regulation of iron uptake in *O. tauri* than in other green lineage organisms [65] or yeast [62].

#### Iron uptake may be mediated by a ZIP-family protein (Ot-Irt1) and a new Fea1-related protein (Ot-Fea1) containing duplicated Fea1 domains

Other important proteins involved in iron uptake and intracellular iron trafficking in plants and in *C. reinhardtii* are the ZIP family transporters IRT and NRAMPs, which are widely conserved from bacteria to humans [15, 23, 26]. We found six genes encoding ZIP family transporters in *O. tauri,* two of which were found to be regulated by iron: ostta06g02310 and ostta16g02300 (Additional file 4: Data Sets 1 and Table 1). The transcript abundance of ostta06g02310 was very low (RNK of 5), and thus the product of ostta16g02300 (733 RNK), which we denote Ot-Irt1, is likely to be the only ZIP protein playing a significant role in iron metabolism in *O. tauri* (Additional file 11: Figure S9 and Table 1). This protein is predicted to be located at the plasma membrane and, interestingly, contains the motif GHGHGHGHGHG, which is similar to the iron-binding motif found in *A. thaliana* IRT1 (PHGHGHGHGP) [25]. Moreover, Ot-Irt1 is more enriched in His residues than At-IRT1 (31 H for 470 amino acids) and contains two additional His-rich motifs in the hydrophilic parts of the protein that are not present in At-IRT1 (Additional file 11: Figure S9), suggesting higher iron-binding capacity of Ot-Irt1 as compared to other IRT proteins in the green lineage. The corresponding gene was particularly strongly induced in the middle of the day after prolonged adaptation to iron deficiency (condition 2), consistent with our previous observation that the ferrous iron uptake capacity of *O. tauri* cells peaks in the middle of the day [9].

As we showed experimentally that *O. tauri* has iron uptake systems for both ferric and ferrous iron and that these systems are induced under iron starvation (Fig. 3), it is reasonable to assume that genes encoding components of these iron uptake systems would be among those most strongly induced by iron limitation in experimental condition 1 and/or condition 2 (Additional file 4: Data Sets 1). One of the most strongly induced genes in all iron-deficient conditions and one of the most strongly expressed genes overall (RNK of 6,521) was ostta18g01980 (Table 1). HMM-based comparison clearly indicated that this protein was related to the *C. reinhardtii* Fea1/2 proteins (Additional file 4: Data Sets 1). This newly discovered *O. tauri* gene (Ot-*FEA1*) has a duplicated Fea1 domain, a signal peptide (predicted by Psort II) and a C-terminal transmembrane helix (predicted by TMpred) (Additional file 12: Figure S10). Interestingly, the N-terminus of Ot-Fea1 is unrelated to Fea1 itself, and this N-terminal region, which is absent from Cr-Fea1, may confer properties different from those of the “regular” Fea proteins of *C. reinhardtii* (Additional file 12: Figure S10B). Moreover, the Ot-Fea1 protein contains several motifs thought to play a key role in iron transport by fungal Ftr1 proteins [22]: R/K-E/D-X-X-E and R/K-E-X-X-E/D. Ot-Fea1 contains three such motifs, which are absent in CrFea1 (Additional file 12: Figure S10A).

**Fig. 4:**
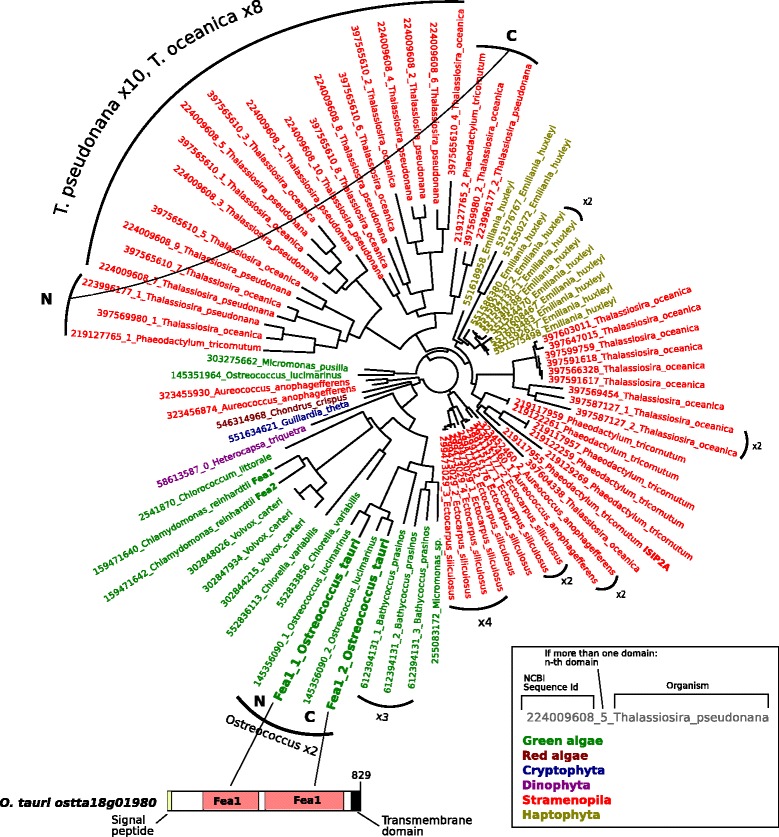
Phylogeny of Ot-Fea1. The newly discovered *O. tauri* gene (ostta18g01980) has a duplicated Fea1 domain (supported by the Hhpred program), a signal peptide (predicted by Psort II) and a C-terminal transmembrane helix (predicted by TMpred). A phylogeny of homologous Fea1 and Isip2a domains from algal proteins is shown. The ostta18g01980 N- and C-terminal domains are monophyletic with the Fea1 domains of other green algae. The multiplication of the Fea1 domain clearly occurred several times independently and even number of domains, from 2 to 10 is generally detected

**Fig. 5:**
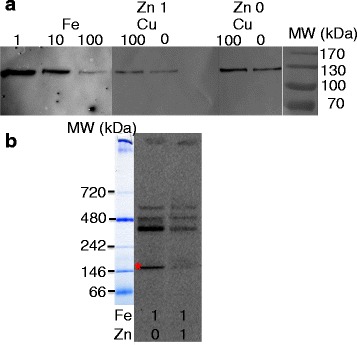
Fe and Zn-dependence of OtFea1 expression, and OtFea1 iron band on native PAGE. **a** *O. tauri* cells were grown for one week in Mf medium containing either 1, 10 or 100 nM Fe (as ferric citrate) or in Mf medium containing 1 nM Fe and different concentrations of Cu (0 or 100 nM CuSO_4_) and Zn (0 or 1 μM ZnCl_2_). Whole-cell extracts were prepared as described in the methods, and proteins (25 μg/lane) were separated by SDS-PAGE before immunoblotting with an anti-OtFea1 primary antibody. **b** Autoradiograph showing the main iron bands after short-term iron loading of the cells and protein separation by native PAGE. *O. tauri* cells were grown for five days in Mf medium containing 1 nM Fe and either 0 or 1 μM Zn, and then incubated for 3 h with 5 μM ^55^Fe(III)-citrate as described in the methods. Whole-cell extracts were obtained and subjected to native PAGE (25 μg/lane). After autoradiography, iron-containing bands were analyzed by mass spectrometry. OtFea1 was found in the band indicated by a star (*)

The role of Fea1 in iron uptake by *C. reinhardtii* remains a matter of debate [2, 46, 51], but we recently described a new protein with a C-terminal Fea1 domain (Isip2a, which also contains two of the motifs mentioned above) that plays an important role in ferric iron uptake by diatoms [49]. A phylogeny of homologous Fea1 and Isip2a domains from algal proteins is shown in Fig. 4. The Ot-Fea1 N- and C-terminal domains are monophyletic with the Fea1 domains of other green algae. Multiplication of the Fea1 domain has clearly occurred on several independent occasions, with the detection of an even number of domains ranging from 2 to 10 (Fig. 4). Mass spectrometry analysis of iron-containing proteins in *O. tauri* led to the identification of Ot-Fea1 with a very good score as one of the proteins that are loaded with iron in *O. tauri* cells (Fig. 5 and Additional file 13: Figure S11). Based on this finding and those of our previous study on Isip2a [49], we suggest that Ot-Fea1 plays a major role in iron uptake (probably in the ferric form) by *O. tauri*. The levels of this protein were also correlated with the effects of different growth conditions on iron uptake: protein abundance was maximal in cells grown in iron-deficient conditions, and zinc deficiency (more than copper) also increased Ot-Fea1 levels (and the level of iron associated with the Ot-Fea1 band on native gel), consistent with an effect of zinc on iron uptake (Fig. 5).

#### Iron is mostly stored in the form of low-molecular weight compounds, rather than ferritin

Ferritins probably play different roles in *C. reinhardtii* and *O. tauri* and are not regulated in the same way in these two species [10, 43]. The fundamental difference between the roles of the ferritin proteins in *C. reinhardtii* and *O. tauri* is highlighted by the different conditions promoting ferritin iron loading in the two organisms: in *C. reinhardtii* the major ferritin, Fer1, displayed maximal loading with ^55^Fe in iron-deficient cells, whereas the *O. tauri* ferritin (ostta02g03400) was more strongly loaded with ^55^Fe in cells maintained in iron-rich medium. Moreover, the total iron content of *O. tauri* ferritin was much lower than that of *C. reinhardtii* ferritin (Additional file 14: Figure S12). This major difference probably accounts for the fact the major ferritins of *C. reinhardtii* and green plants behave like most ferritins, as iron storage proteins protecting the cell against oxidative stress and storing iron for later use [12, 14, 43], whereas the *O. tauri* ferritin behaves like a protein involved in intracellular iron recycling [10], as does the ferritin of the diatom *Pseudonitzschia multiserie*s [55]. As previously reported [47], the ferritin gene displayed circadian regulation, with expression levels being highest at the end of the night [10]. This strongly expressed gene (1167 RNK) was also upregulated (during the night only) by prolonged growth at low iron concentrations (Table 1 and Additional file 4: Data Sets 1), consistent with its role in recycling iron from iron-containing proteins expressed during the day [10]. This result contrasts with observations for *C. reinhardtii*, in which both the ferritin genes, *FER1* and *FER2,* are strongly induced by iron starvation during the day [72]. The authors suggested that ferritins, together with the manganese superoxide dismutase, could protect the cells against ROS, which may become more abundant when PSI function is compromised [72]. In *O. tauri,* the copper/zinc superoxide dismutase (ostta01g04820) was repressed by long-term iron starvation, particularly during the day (Additional file 4: Data Sets 1; and see below: Stress and redox response).

As the main role of *O. tauri* ferritin relates more to iron redistribution than to iron storage [10], it remains unclear how iron is stored in this organism. The vacuole is the main iron-storage compartment in several organisms (particularly yeasts lacking ferritin) [58] and a protein homologous to the yeast vacuolar iron importer Ccc1 is found in plants (VIT1) [33]. In *C. reinhardtii*, the corresponding gene was unexpectedly shown to be upregulated by iron deprivation [72]. We found a homolog in *O. tauri* (Ot-*VIT1:* ostta07g00660) that was repressed by iron starvation (Additional file 5: Data Sets 2), consistent with a role of the vacuole in iron storage. However, it is not clear whether *O. tauri* actually has a vacuole, due to the small size of this eukaryote. Numerous small vesicles have been described in *O. tauri* [28] and the storage compartment could thus be a specialized small vacuolar structure related to the polyphosphate bodies (acidocalcisomes) containing polyphosphate and metals described in *C. reinhardtii* [45]. This hypothesis seems probable, because a gene encoding a typical vacuolar-type H^+^-pyrophosphatase found in acidocalcisomes [39] is present in the *O. tauri* genome (ostta01g00980), and this gene was induced by iron deprivation, particularly during the night (Table 1). Conversely, the inorganic phosphate transporter (ostta02g02460) was strongly downregulated by iron limitation at almost all time points (Table 1 and Additional file 4: Data Sets 1), which suggests the existence of a strong relationship between iron and phosphorus metabolism in *O. tauri,* as demonstrated in land plants [53]. Iron could thus be stored in acidocalcisome-like vesicles, possibly as polyphosphates, rather than ferritin, as suggested by an analysis of the intracellular distribution of iron. When whole extracts of ^55^Fe-loaded *O. tauri* cells were subjected to fractionation by gel filtration (FPLC), the major iron peak was associated with low-molecular weight compounds (<17 kDa; Additional file 15: Figure S13). We are currently investigating this issue of ferritin-independent iron storage in *O. tauri*.

### The adaptation of cells to iron limitation involves iron-sparing mechanisms, stress responses, and general metabolic remodeling with concomitant recruitments for zinc-containing regulatory proteins, as a function of the day/night cycle

#### Iron-sparing response, tetrapyrroles and lipids

One of the expected responses of cells to iron starvation is to decrease the amounts of iron-containing proteins not strictly required for cell viability. This was observed to some extent: one of the major sinks for iron concerns the use of nitrate as the nitrogen source. A repression of genes encoding a nitrate transport protein (ostta10g00950), nitrate reductase (ostta10g00920) and ferredoxin-nitrite reductase (ostta10g00930) was observed in both the early and later adaptive responses to iron limitation (Table 1 and Additional file 4: Data Sets 1). In parallel, a RWP-RK transcription factor (ostta14g01760) likely involved in the transcriptional response to nitrate [17] was strongly induced in all conditions, and a zinc GATA factor possibly involved in nitrogen metabolism (ostta07g03410) was also strongly induced (Table 1). Conversely, the ammonium transporter (ostta12g00300) was induced during the day in cells adapted to iron limitation (Additional file 4: Data Sets 1), possibly to compensate for the lower levels of nitrate transport. Repression was also observed in all conditions for the gene encoding the [2Fe-2S] iron-sulfur cluster protein adrenodoxin, ferredoxin-like protein (ostta11g0770), and for key enzymes involved in the synthesis of hydroxyproline-rich glycoproteins (several di-iron prolyl-4-hydroxylases) and in lipid metabolism (di-iron-containing fatty acid desaturases, sterol desaturase, fatty aldehyde decarbonylase/acyl-ACP reductase etc.) (Table 1 and Additional file 4: Data Sets 1). Four 2OG-Fe(II) oxygenase (prolyl-4-hydroxylase)-encoding genes (ostta04g02880, ostta04g00640, ostta16g00350 and ostta11g00880) were globally repressed in iron-deficient growth conditions (Additional file 4: Data Sets 1), with probable consequences for cell surface properties, because hydroxyproline-rich glycoproteins are the major proteinaceous components of the cell walls in green algae [21]. The downregulation of fatty acid desaturases would also be expected to change the lipid composition of membranes (see below). Other iron-containing proteins vital for cell division (ribonucleotide reductase ostta08g00560, JmjC domain cupin-like protein ostta15g00920) were not downregulated by iron deprivation, and a global upregulation of these proteins was even observed (Additional file 4: Data Sets 1), reflecting the optimization of iron use/redistribution to high-priority iron-proteins during iron limitation.

**Fig. 6:**
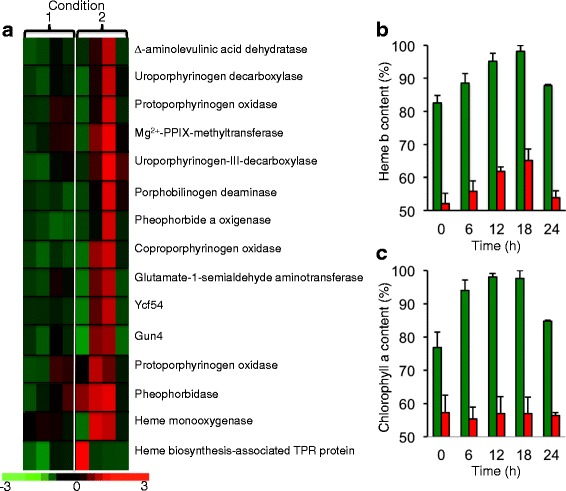
Cell response to iron deprivation in terms of tetrapyrrole metabolism. **a** Clustering of data showing differential expression of the genes involved in tetrapyrrole synthesis and degradation in two sets of experimental conditions. Results are expressed (according to the color scale) in log base 2, as logFC [−Fe]/[+Fe]. **b** Heme *b* content of cells over a 24-h period, after one week of adaptation to iron-rich (*green*) or iron-poor (*red*) conditions (experimental condition 2). **c** Chlorophyll *a* content of cells over a 24-h period, after one week of adaptation to iron-rich (*green*) or iron-poor (*red*) conditions (experimental condition 2)

As stated above, the long-term adaptation of cells to iron limitation was largely determined (in terms of genome-wide patterns of expression) by day/night cycles. Thus, most genes were neither fully induced nor fully repressed, instead being up- or downregulated at different points in the day/night cycle. The genes encoding proteins involved in tetrapyrrole synthesis provide a typical example. Unsurprisingly, iron deprivation leads to some chlorosis in *O. tauri* (although much less pronounced than that observed in acetate-grown *C. reinhardtii* cells), and both the chlorophyll *a* content and the heme *b* content of the cells were significantly decreased by iron starvation (Fig. 6). The decrease in chlorophyll content could result from both a decrease in synthesis (because magnesium-protoporphyrin IX monomethyl ester cyclase is a di-iron enzyme) and an increase in degradation: chlorophyllide *a* oxygenase (ostta07g02450), another iron-containing enzyme involved in chlorophyll *b* synthesis, was slightly repressed, whereas the pheophorbidase gene ostta01g02150 (which is involved in chlorophyll degradation) was strongly upregulated under iron starvation, as was the gene encoding pheophorbide *a* oxygenase (ostta12g01390), a key regulator of chlorophyll catabolism [29] (Table 1 and Additional file 4: Data Sets 1). The decrease in heme content probably resulted from the induction of heme oxygenase (ostta10g01650) under conditions of iron deprivation (Table 1), which probably made a significant contribution to the iron-sparing response by allowing the cells to recycle iron from heme. The *GUN4* gene, encoding a major regulator of tetrapyrrole synthesis [38], was also strongly affected by iron nutrition conditions, as a function of the day/night cycle, and the transcription pattern of this gene was consistent with the general transcription pattern for all genes involved in heme and chlorophyll biosynthesis (Fig. 6): in conditions of rapid iron depletion (condition 1) these genes were slightly repressed during both the day and night (rapid iron-sparing response, accompanied by a slight corepression of heme oxygenase), but when the cells were allowed to adapt to low iron conditions for several days (condition 2), the transcription profile of these genes responded to the day/night cycle, with a peak of transcription at the start of the night (accompanied by the induction of heme oxygenase), consistent with the actual concentrations of heme *b* in the cells over a 24-h period (Fig. 6). Thus, although the global level of transcription (over a 24 h period) of genes involved in tetrapyrrole synthesis was decreased by iron deprivation, and the total amount of heme was decreased (Fig. 6), the cells maintained the day/night regulation pattern of these genes as in iron-replete cells, probably to optimize cell functions and synchronize these functions with the day/night redistribution of iron [10] and the redistribution of iron resulting from long-term iron limitation. By contrast, the day/night variation in chlorophyll *a* content was abolished under low-iron conditions (Fig. 6).

The main iron-containing fatty acid desaturases (ostta03g03040, 702 RNK; ostta05g00100, 556 RNK, Table 1) were globally repressed, probably as an iron-sparing response, although gene regulation remained largely responsive to the day/night cycle. As a result, the lipid composition of iron-deficient cells would be expected to shift towards more unsaturated fatty acids, as shown in *C. reinhardtii* [71]. An extensive analysis of the changes in lipid composition induced by iron deprivation will be presented elsewhere.

#### Stress and redox response

As shown in Fig. 2 and Table 1, the functional category “stress and redox response” was the third largest category of genes upregulated in response to short-term iron starvation (8 %, after “Regulatory” and “Unknown” genes), and the fourth (7 %) largest category of genes up-regulated during the long-term adaptation of cells to iron-deficient conditions.

In *C. reinhardtii*, iron deprivation results in an increase in antioxidant defense through the induction of genes involved in ascorbate synthesis (namely *VTC2* and *MDAR1*) [72]. As indicated above, the homologous genes were not regulated by iron in *O. tauri*. Moreover, most of the “stress and redox response” gene products induced by iron deprivation in *O. tauri* have homologs not induced by iron deprivation in *C. reinhardtii*, with some of these homologs even being repressed in such conditions (Additional file 4: Data Sets 1). The stress response to iron deficiency therefore appears to be very different between these two organisms, reflecting fundamental differences in their iron metabolism (see the “Stress and redox response” functional category in Additional file 4: Data Sets 1). The early response to iron deficiency in *O. tauri* (condition 1) involves the recruitment of Hsp20-like and other molecular chaperones (ostta18g01945, ostta08g01350), and the induction of a peroxiredoxin (alkyl hydroperoxide reductase; ostta18g00200), but not of the copper/zinc superoxide dismutase (Table 1 and Additional file 4: Data Sets 1). After long-term adaptation to iron limitation (condition 2), the most strongly upregulated genes were found to be those involved in DNA repair (including Rad50, Rad51, Rad3, Rad9, a GIY-YIG nuclease ostta16g00515, and other DNA repair genes/proteins) and genes involved in thiol-based redox processes, such as a thioredoxin fold-like selenocysteine (ostta10g01410), a pyridine nucleotide-disulfide oxidoreductase (ostta18g00200), thioredoxins/glutaredoxins (ostta10g01420 and ostta10g01310) and a glutathione-S-transferase (ostta01g02840) (Table 1 and Additional file 4: Data Sets 1).

Surprisingly, *Ostreococcus* has no gene for phytochelatin [52], but a tesmin-like methallothionein gene was induced, particularly during the night (ostta09g04220). The product of this gene may be involved in heavy metal detoxification (Table 1).

#### Metabolism

About 20 % of the selected genes (both conditions) encoded components of general metabolism (including carbohydrate metabolic processes, carbon metabolism, the Calvin cycle and photosynthesis and other metabolic processes). The rate of photosynthesis, as determined by oxygen production, in iron-deficient cells was half that in iron-rich cells (about 16 and 30 nmoles O_2_/min/million cells, respectively), and the transcription of some key genes involved in the photosynthetic electron chain was significantly downregulated (see the functional category “Photosynthesis” in Additional file 4: Data Sets 1): the most strongly repressed gene was ostta10g02860, encoding a flavin amine oxidase involved in the synthesis of carotenoids (phytoene dehydrogenase or desaturase), and the non-mevalonate 1-deoxy-d-xylulose-5-phosphate (DOXP) pathway for the biosynthesis of plastid isoprenoids was probably also repressed through strong repression of the 1-deoxy-D-xylulose-5-phosphate synthase gene (ostta07g04370). Genes encoding components of PSI and PSII were also globally repressed after prolonged growth under iron limitation, reflecting compromised photosystem reaction centers and low rates of photosynthetic electron transport, even though the transcriptional profiles of these genes remained largely determined by the day/night cycles under iron-deficient conditions (Table 1 and Additional file 4: Data Sets 1). However, some photosynthetic genes, including the genes encoding the PSII reaction center PsbM (ostta12g00420), the PSII oxygen-evolving complex PsbQ (ostta16g01620) (both Psb genes being induced during the night), subunit B of the cytochrome *b*_*6*_*f* complex (PetN, ostta12g00550) and violaxanthin de-epoxidase (ostta09g01160), were upregulated, probably indicating an increase in the ratio of PSII to PSI complexes and an increase in non-photochemical quenching capacity, as observed in other iron-starved photosynthetic organisms [1].

Nothing is known about mitochondrial iron metabolism in *O. tauri*. The mitochondrial [Fe-S] cluster assembly machinery was probably impaired by iron limitation, because a gene homologous to *ATM1* (ostta13g00830), encoding a key component for [Fe-S]-mediated electron transport was slightly, but significantly repressed in all conditions of iron limitation (Additional file 5: Data Sets 2), and the LYR family protein ostta06g02630, which is homologous to the yeast Isd11 protein (a scaffold protein required for mitochondrial [Fe-S] cluster assembly), were repressed after prolonged iron deprivation, particularly during the day (Table 1 and Additional file 4: Data Sets 1). These data suggest that the adaptation of *O. tauri* to iron limitation involves a decrease in respiratory functions, consistent with the high iron requirement of the respiratory chains. Interestingly, the gene encoding the alternative oxidase (ostta16g0930) was upregulated during the night in long-term adapted cells (as previously shown in the diatom *P. tricornutum* [1]), potentially limiting ROS production by the impaired respiratory chain (Additional file 5: Data Sets 2).

In addition to reprogramming nitrogen metabolism, which could be considered to be an iron-sparing response, a number of key genes involved in carbon metabolism were up- or downregulated by iron starvation. An extensive description of iron-dependent metabolism remodeling is beyond the scope of this manuscript but will be provided elsewhere.

#### Regulation

**Fig. 7:**
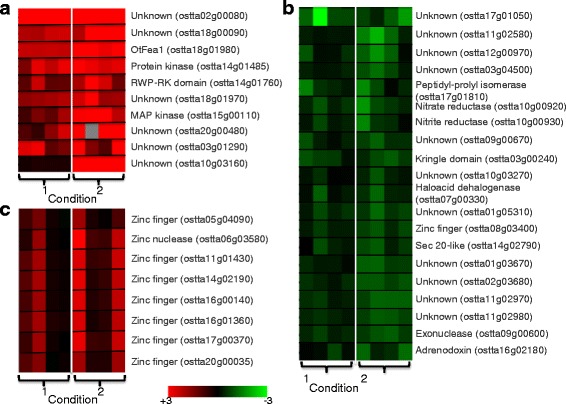
Clusters of genes globally induced (**a**) or repressed (**b**) by iron starvation and cluster of genes encoding zinc regulatory proteins. We performed a clustering analysis (using the k-means method) on the complete dataset; very few genes were upregulated in all conditions (experimental conditions 1 and 2, dark/light): these genes are grouped in cluster A. Similarly, genes downregulated in all conditions are grouped in cluster B. One cluster (cluster **c**) grouped genes encoding zinc regulatory proteins. Results are expressed (according to the color scale) in log base 2 as logFC [−Fe]/[+Fe]

Most of the genes either up- or downregulated by iron in both sets of growth conditions were either “Regulatory” or “Unknown”, and the “regulatory” functional category accounted for about 25 % of the genes analyzed (Fig. 2). Most of the regulatory genes were directly or indirectly related to replication, transcription or translation and to ribosomal functions, but a lot of posttranslational mechanisms were also involved in the adaptation of cells to iron limitation. These mechanisms included phosphorylation/dephosphorylation, chaperoning and maturation, protein sorting and vesicular trafficking, and glycosylation (see the “Regulatory” functional category in Additional file 4: Data Sets 1). Nothing is known about the regulation of iron-related responses in *O. tauri*. In *A. thaliana* and *C. reinhardtii*, several basic helix-loop-helix (bHLH) transcription factors and an E3 ubiquitin ligase are involved in transcriptional regulation of the iron regulon, in a complex network of iron sensing and signaling [63, 72, 75]. Myb-like transcription factors are also involved in regulating iron uptake and storage in plants and in *C. reinhardtii* [64, 72]. There is one bHLH protein homologous to *A. thaliana* FIT1 (Fe-deficiency-induced transcription factor 1) [5] in *O. tauri* (ostta14g01990), but the expression of the corresponding gene was not significantly affected by iron limitation (Additional file 5: Data Sets 2), and the network of bHLH transcriptional regulators in the green algal lineage does not seem to be conserved in *O. tauri*. However, several Myb-like transcription factors were downregulated by iron limitation (Additional file 4: Data Sets 1), and the transcription of many other transcription factors was induced/repressed in response to iron limitation (see the “Regulatory” functional category in Additional file 4: Data Sets 1). In *C. reinhardtii*, genes directly involved in reductive iron uptake are coordinately coregulated (together with *FEA1*), and it has been suggested that regulation involves the activation of genes under conditions of low iron availability rather than repression at high iron concentrations [2]. We were unable to identify the equivalent of an “iron regulon” in *O. tauri*, *i.e*., an ensemble of genes directly involved in iron metabolism and primarily regulated by iron at the transcription level. It is however possible that the apparent lack of a clear iron regulon simply results from our lack of knowledge: as shown in Fig. 7a, there were very few genes that were upregulated by iron deprivation regardless of the day/night cycles and of the experimental conditions (like Ot-Fea1), but most of these genes encode unknown proteins. The same applied for genes downregulated by iron deprivation: very few genes were downregulated regardless of the day/night cycles and of the experimental conditions, and most of these genes have unknown function (Fig. 7b). In the overwhelming number of cases, iron controlled transcription differentially, according to the day/night cycles and to the experimental conditions (short-term/long-term response). The mechanisms of transcriptional regulation involved in the whole-cell response to iron limitation are, therefore, probably multifactorial and complex.

There was an obvious transcriptional co-activation of zinc-containing regulatory proteins (Fig. 7c). These zinc-containing regulatory proteins were either ZF_C2H2 zinc fingers (C2H2 type) or PRLI-interacting factor K zinc fingers with no homolog in *C. reinhardtii*, and the corresponding genes displayed strikingly similar patterns of regulation (in condition 1: induction only during the day; in condition 2: induction at the start of the day and the end of the night) (Fig. 7 and Additional file 4: Data Sets 1). As shown above, zinc had strong effects on iron uptake that depended on the growth conditions. Conversely, we show here that iron deprivation results in the transcriptional activation of numerous genes encoding zinc-containing regulation factors. Transcriptional effects thus probably underlie at least part of the connection between zinc and iron in *O. tauri*, through the recruitment of zinc proteins under conditions of low iron concentration. Such zinc regulation factors may be required for full activation of the Ot-Fea1-dependent iron uptake system under iron deprivation, potentially accounting for the incomplete induction of iron uptake in zinc-deprived cells.

## Discussion

Our work reveals tight coordination of iron metabolism with diurnal cycles in *O. tauri*. This observation generalizes our previous findings that intracellular iron is cycled from the main iron proteins (during the day) to ferritin (during the night) [10], and that cell iron uptake capacity also varies over day/night cycles [9]. A similar dependence of iron metabolism on the circadian clock has also been reported in higher plants [69].

*O. tauri* rapidly responds to decreased iron nutrition by inducing the uptake of both ferric and ferrous iron, but (unlike *C. reinhardtii*) the ferrireductase activity of the cells is very low and is not induced by iron deprivation. The earlier induction of the iron uptake systems in *O. tauri* compared to *C. reinhardtii* in response to iron starvation may reflect differences in the role of ferritin in the two organisms: ferritin does not behave like an iron-storage protein in *O. tauri* [10], and its weaker iron storage capacity may result in more rapid iron starvation than in *C. reinhardtii* cells, which can use ferritin as an iron store. However, the rapid induction of iron uptake activities in *O. tauri* is not fully reflected by transcriptomic data: whereas *C. reinhardtii*, coordinately induces genes encoding ferrireductase, ferrooxidase and iron permease proteins (components of the reductive iron uptake system and ZIP family transporters) in response to iron deprivation, we could only identify one gene (Ot-*FEA1*) directly involved in iron uptake that was primarily induced by iron deprivation in all conditions (day/night, short term/long term response) in *O. tauri*. However, it is always possible that some genes unaffected by iron at the transcriptional level could be regulated at the post-transcriptional level. The difference in the transcriptional response to iron limitation in *O. tauri* and *C. reinhardtii* could result from several factors.

1. To our knowledge, the response of *C. reinhardtii* to changes in iron nutrition was never investigated according to the day/night cycles. Such a study would perhaps also reveal a strong dependence of the cell response to iron limitation to the diurnal parameter.
2. The mechanisms of iron uptake are probably very different in both organisms, since *O. tauri* lacks the classical components of reductive iron uptake system. Two genes encoding putative ferrireductases (cytochrome b561) were induced lately and are unlikely to participate in reductive iron uptake in *O. tauri*. As shown in yeast and plants [18, 41, 74], intracellular iron use involves many different ferrireductases, and cytochrome b561 may be involved in supplying ferrous iron to the chloroplast, as suggested in other organisms [30, 72].
3. A lot of genes showing a strong response to iron depletion encode proteins with unknown function in *O. tauri*, which indicates that several pathways of iron uptake and metabolism remain to be described in this organism.

Our work strongly suggests that Ot-Fea1 is directly involved in iron uptake in *O. tauri*. Homologues of the *C. reinhardtii* Fea1/Fea2 proteins have been previously considered in the green algal lineage [7], but Ot-Fea1 is structurally very different than Cr-Fea1 (it cannot be found by simple BLAST search) and presents unique features that make it a good candidate for nonreductive ferric iron uptake in *O. tauri*, as we recently showed for ISIP2a in diatoms [49]. Despite of several attempts, we were not able to generate a ∆*Ot-Fea1* knockout in *O. tauri*, suggesting that the gene is vital for growth. We are currently trying to determine the conditions of Ot-Fea1 iron loading *in vitro* by overproducing this protein in *E. coli* and then purifying it and incubating it with extracts of *O. tauri* cells grown in different conditions.

Ot-Irt1 is another protein that is most probably involved in iron uptake in *O. tauri*, and it differs from other Irt-like proteins of the green lineage by the abundance of His-rich motifs (GHGHGHGHGHG). Grossoehm *et al.* [25] showed that such His-rich sequences (which is further enriched by 2 additional H residues in Ot-Irt1) has a high entropy-driven affinity for Fe^3+^ and might be involved in retaining ferric iron on the cytoplasmic side of the protein, thereby inhibiting further ferrous iron uptake by the extracellular side of the protein. This might be of particular relevance for *O. tauri*, since this organism is probably able to take up iron without prior reduction.

Other components involved in iron uptake by *O. tauri* probably remain to be identified. A better understanding of the mechanisms of iron uptake should account for the much higher efficiency of *O. tauri* cells to concentrate extracellular iron as compared to *C. reinhardtii*: our work shows that *O. tauri* needs much less iron in the medium than *C. reinhardtii* to reach maximal growth rate, although the iron requirement of both organisms is similar. This is the main reason why we propose *O. tauri* as an alternative model to study iron metabolism in the green algal lineage. To date, the transcriptional response to iron limitation in marine micro-algae was mainly investigated in diatoms. Some centric diatoms like *Thalassiosira pseudonana* have genes encoding all the components of a classical reductive iron uptake system similar to that of *C. reinhardtii* [4]. In *T. pseudonana*, Kustka *et al.* [35] showed that the genes encoding plasma membrane ferrireductases (*TpFRE1* and *TpFRE2*) and two very similar iron permeases (*TpFTR1* and *TpFTR2*) were upregulated in response to iron deprivation, while the gene encoding a putative multicopper ferroxidase (*TpFET3*) was not responsive to iron. In contrast, *FET3* and *FTR1* homologues are lacking in the genome of the pennate diatom *Phaeodactylum tricornutum*, which suggests that iron uptake does not involve a ferroxidase-permease complex in this species [35]. The transcriptional response of *P. tricornutum* to iron starvation involves the strong upregulation of *ISIP* genes (Iron Starvation Induced Proteins), among which *ISIP2A*, encoding a protein that concentrates iron at the cell surface and facilitates its uptake [1, 49]. There is thus some similarity between *P. tricornutum* and *O. tauri*, since both species lack the classical components of a reductive iron uptake system (although *P. tricornutum*, but not *O. tauri* has an inducible ferrireductase activity) and both species respond to iron deprivation by inducing proteins that are phylogenetically related (through the Fea1 domain): Isip2a (for *P. tricornutum*) and Ot-Fea1 (for *O. tauri*).

Besides iron uptake, the transcriptional response of *O. tauri* to iron limitation differs from that of other chlorophytes (*C. reinhardtii*, *A. thaliana*) by several important features, especially related to the stress response. An increase in antioxidant mechanisms in response to iron limitation has been demonstrated in various plants and algae [59, 72], and a key defense response has been shown to be mediated by ascorbate in *C. reinhardtii*, in which iron limitation results in a 10-fold increase in cell ascorbate content [72]. At the same time, the response of *C. reinhardtii* to iron deprivation involves induction of the Cu/Zn superoxide dismutase and of both ferritins Fer1 and Fer2 [72]. We show here that the response of *O. tauri* to iron deprivation is completely different: transcription of key genes involved in ascorbate synthesis and of the gene encoding Cu/Zn superoxide dismutase is unaffected by the iron status in *O. tauri*. As shown in other organisms [54], peroxiredoxin (induced by iron deprivation in *O. tauri*) may therefore be the main scavenger of endogenous hydrogen peroxide in *O. tauri*. Ferritins also behave very differently in *O. tauri* and *C. reinhardtii*, with respect to iron-dependent transcription of the corresponding genes, but also regarding the conditions promoting iron loading of the proteins (see Conclusions).

## Conclusions

There is increasing evidence to suggest that several marine microalgae have iron uptake systems different from those in terrestrial organisms, involving for example, the nonreductive uptake of inorganic ferric species, as an adaptation to the extreme scarcity of iron prevailing over vast ocean regions. *O. tauri* could therefore be used as an alternative model alga (to *C. reinhardtii*, the model freshwater alga) for studying iron metabolism in unicellular marine photosynthetic organisms. Our results show that iron metabolism differs considerably between these two species. *O. tauri* is much more efficient than *C. reinhardtii* at taking up iron from media with low iron content and it can grow with as little as one hundredth the amount of iron required by *C. reinhardtii*. This organism must, therefore, have powerful iron uptake systems. The novel protein Ot-Fea1 seems to be involved primarily in iron uptake by *O. tauri*.

One striking feature of *O. tauri* iron metabolism is its tight dependence on the day/night cycle. Among the genes displaying differential expression with a log ratio >1.5 for at least one time point after adaptation of the cells to iron limitation (condition 2), only 7 % were induced during both the day and the night (4 time points). The transcriptional response to iron limitation was therefore highly dependent on the diurnal cycle. This diurnal regulation probably reflects the intracellular recycling of iron between iron-containing proteins and ferritin [10]. Regulation of the ferritin gene is multifactorial and ferritin itself has a regulatory function, possibly related to iron sensing. The ferritin gene is under circadian regulation [47] and is also regulated by iron in a complex manner: in short-term experiments we showed that high iron concentration in the medium resulted in larger amounts of the translation product, in experiments using a ferritin-Luc reporter gene [10]. However, we also show here that the ferritin gene was upregulated only during the night in conditions of long-term iron limitation. Moreover, ferritin is itself involved in the regulation of its own gene, as we were able to show that circadian regulation was abolished for a ferritin-luc homologous recombinant (*i.e.,* when the ferritin gene was expressed with a tag that prevented the assembly of a functional ferritin multimer). Ferritin is also directly or indirectly involved in the regulation of iron uptake (because iron uptake rate was low in a ferritin knockout strain) [10]. These data suggest that iron homeostasis is orchestrated around ferritin in *O. tauri*. Such a mode of regulation of iron homeostasis has to our knowledge never been described previously, but could be widespread in marine microalgae: recent work in *Pseudonitzschia multiserie*s showed that the ferritin of this marine diatom was more involved in iron redistribution than in iron storage [55].

Another striking feature of *O. tauri* iron metabolism is its connection with zinc metabolism. Such a strong iron/zinc connection has not been described in *C. reinhardtii*, and could be related to the particular mechanisms of regulation of iron metabolism in *O. tauri*, involving the recruitment of many zinc-containing proteins.

We currently know very little about the molecular mechanisms of iron uptake and homeostasis in eukaryotic phytoplankton, and this lack of knowledge is reflected here, because the genes displaying the highest levels of differential expression as a function of iron nutrition had unknown functions. The very large number of such “pioneer proteins” is another striking feature that emerges from our work.

## Methods

### Strains, cell culture and media

*Ostreococcus tauri* was grown at 20 °C under a 12 h:12 h light (3,000 lux)/dark regime in a filtered modified f (Mf) medium, as previously described [68]. The composition of the Mf medium was as follows: 40 g/l sea salts (Sigma; composition: 19.29 g/l Cl^−^, 10.78 g/l Na^+^, 2.66 g/l SO_4_^2−^, 1.32 g/l Mg^2+^, 420 mg/l K^+^, 400 mg/l Ca^2+^, 200 mg/l CO_3_^2−^/HCO^3−^, 8.8 mg Sr^2+^, 5.6 mg/l BO^2−^ 5.6, 56 mg/l Br^−^, 0.24 mg/l I^−^, 0.3 mg/l Li^+^, 1 mg/l F^−^); 250 mg/l MOPS (pH 7.3); 2.66 mg/l NH_4_NO_3_; 75 mg/l NaNO_3_; 22.8 mg/l Na_2_SiO_3_ · 5H_2_O; 15 mg/l NaH_2_PO_4_; 1 ml of vitamin stock (20 mg/l thiamine HCl, 1 mg/ml biotin, 1 mg/ml B12); 1 ml of trace metal stock (200 mg/l MnCl_2_ · 4H_2_O, 40 mg/l ZnSO_4_ · 7H_2_O, 20 mg/l Na_2_MoO_4_ · 2H_2_O, 14 mg/l CoCl_2_ · 6H_2_O, 10 mg/l Na_3_VO_4_ · nH_2_O, 10 mg/l NiCl_2_, 10 mg/ml H_2_SeO_3_); and 1 ml of antibiotic stock (100 mg/ml ampicillin sodium and streptomycin sulfate). The Mf medium was buffered with 1 g/l HEPES (pH 7.5). Iron was added as 0.1 μM ferric citrate (1:20) for culture maintenance. *Chlamydomonas reinhardtii* was grown under a 12 h:12 h light (3,000 lux)/dark regime in modified TAP medium. The composition of modified TAP medium was as follows: 2.42 g/l Tris; 25 ml TAP salt solution (15 g/l NH_4_Cl, 4 g/l MgSO_4_ · 7H_2_O, 2 g/l CaCl_2_ · 2H_2_O); 0.375 mL phosphate solution (288 g/l K_2_HPO_4_ 288, 144 g/l KH_2_PO_4_), 1 ml modified Hunter’s trace element solution (50 g/l EDTA disodium salt, 22 g/l ZnSO_4_ · 7H_2_O, 11.4 g/l H_3_BO_3_, 5.06 g/l MnCl_2_ · 4H_2_O, 1.61 g/l CoCl_2_ · 6H_2_O, 1.10 g/l (NH_4_)_6_Mo_7_O_24_ · 4H_2_O). Iron was added as 1 μM ferric citrate (1:20) and copper was added as 6.3 μM copper sulfate for culture maintenance. Different sources of iron were added at various concentrations for particular experiments, as described below.

Determination of growth rates of *O. tauri* and *C. reinhardtii* as a function of extracellular Fe and Cu: the cells were grown for one week in low-copper low-iron medium (for *O. tauri*: Mf medium with no added copper and 10 nM ferric citrate; for *C. reinhardtii*: modified TAP medium containing 10 nM CuSO_4_ and 100 nM ferric citrate), washed once in iron-free and copper-free medium, and then dispensed (at about 10^6^ and 5 x 10^3^ cells/ml for *O. tauri* and *C. reinhardtii,* respectively) into the wells of a 96-well microtiter plate containing 200 μl/well Mf (*O. tauri*) or modified TAP (*C. reinhardtii*) medium, containing a range of Fe (ferric citrate) and Cu (CuSO_4_) concentration. Growth was followed everyday by flow cytometry, and maximal growth rates were determined from the growth curves.

Determination of intracellular iron content: the cells were precultured as above and then dispensed (at about 10^6^ and 5 x 10^3^ cells/ml for *O. tauri* and *C. reinhardtii,* respectively) into the wells of a 6-well microtiter plate containing 5 ml/well Mf (*O. tauri*) or modified TAP (*C. reinhardtii*) medium, containing a range of ^55^Fe concentration, as either ferric citrate (1:20) or ferric EDTA (1:100). The cells were then washed as described below (^*55*^*Fe uptake*). Results were normalized to the cell volumes according to the following parameters: *O. tauri* and *C. reinhardtii* cells were considered as spheres with a ratio of 0.85 μm and 3.2 μm respectively.

### Generation of samples for RNASeq analysis

The experimental conditions are illustrated in Additional file 1: Figure S1. In one set of experiments (condition 1), we grew cells to the exponential phase under a 12 h:12 h light–dark regime in a medium containing 0.1 μM Fe(III)-citrate, and, at *t* = 0 we added excess of iron (1 μM) to half the cultures, and 2 μM of the iron chelator DFOB to other half (as we previously showed that *O. tauri* cannot use the siderophore FOB [68]. These experiments were performed in the light and in the dark: cells were grown in two growth chambers in opposite phases, under a 12 h:12 h light–dark regime, and excess iron/DFOB was added at dawn and dusk. Cells were harvested 3 h and 6 h after the addition of iron/DFOB in the light and in the dark. In a second set of experiments (condition 2), the cells were grown for one week in SOW medium, and the cells were then shifted (at a density of 10^6^ cells/ml) for AQUIL medium containing either 54 nM ferric EDTA (low iron conditions) or 1080 nM ferric EDTA (high iron conditions). After one week of growth under a 12 h:12 h light/dark regime, cells were harvested at different moment of the day/night cycle: 3 h, 9 h (day), 15 h, and 22 h (night) after dawn. All experiments were performed in triplicate (biological triplicates, meaning that 3 independent cultures were used for each set of conditions).

### RNA extraction, sequencing

RNA was extracted as described by Moulager *et al*., [50]. All manipulations were carried out on ice. The 48 RNA samples were subjected to high-throughput transcriptome sequencing with an Illumina HiSeq™ 2000 (Fasteris company, Switzerland).

### Bioinformatics analyses

#### Analysis of raw sequencing data

The general protocol used for the analysis of raw sequencing data (FASTQ files) is presented in Additional file 2: Figure S2. FASTQ files comprise the sequencing results (reads). Step 1 consisted in controlling the quality of sequencing results using FASTQC (http://www.bioinformatics.babraham.ac.uk/projects/fastqc/) and CUTADAPT programs (https://cutadapt.readthedocs.org/en/stable/). Step 2 consisting in mapping the reads on the *O. tauri* CDS sequences available in the ORCAE database [66], using the BOWTIE program [37]. Step 3 Mapping outputs were next converted in SAM and BAM files with SAMTOOLS programs (http://samtools.sourceforge.net/) for visual inspection with IGV program [61]. Step 3 consists in calculating the number of reads mapped on each CDS sequence in each condition, using the BEDTOOLS program (http://bedtools.readthedocs.org/en/latest/). Numbers of reads were normalized applying the DEseq program [3].

#### Functional annotation of genes

A systematic process combining information from multiple web resources was applied for inferring gene function. This process consisted in searching the ORCAE database (http://bioinformatics.psb.ugent.be/orcae/), looking for predicted domains for each gene product (homology detection and structure prediction by HMM-HMM comparison (http://toolkit.tuebingen.mpg.de/hhpred) and Pfam database (http://pfam.xfam.org/)). To assess whether any of the functions were observed in gene clusters at a frequency greater than that expected by chance, p-values were calculated using hypergeometric distribution as described in [11].

#### Orthology *assignment*

Orthology relationships were inferred between *O. tauri* and *C. reinhardtii* using the INPARANOID program [60].

#### Phylogenetic analysis of Ot-Fea1

We first collected a dataset of known Fea and Isip homologs (*C. reinhardtii* Fea1 and Fea2 and *P. tricornutum* Isip2a and Isip2b), aligned them with Mafft (−−maxiterate 1000 --localpair options) [32] and trimmed the alignment by hand. The resulting alignment was then used for iterative searches of the nr protein database with Jackhmmer (e-value cutoff 1e-5) until the searches converged. Sequences displaying similarity to the original alignment were extracted and, using Mafft (−−maxiterate 1000 --localpair options). The alignment was trimmed with BMGE (matrix BLOSUM30 and block size 1) and a phylogeny was constructed with Phyml (WAG substitution model) [27].

### Determination of growth rate

Cell growth was determined with a flow cytometer (BD Accury C6), as described previously [9].

#### Ferrireductase activity

The whole-cell ferrireductase activity expressed by microalgae was measured as previously described [68] with Fe(III)-EDTA as the iron source. The cells (50–500 million cells per mL) were incubated in Mf medium (*Ostreococcus tauri*) or TAP medium (*Chlamydomonas reinhardtii*) at 20 °C in the dark in the presence of iron (0.5 mM) and BPS (2 mM) and aliquots were collected at intervals and centrifuged (10,000 × *g*, 5 min) for spectrophotometric determination of the amount of Fe^2+^(BPS)_3_ at 535 nm.

#### ^55^Fe uptake

Iron uptake by microalgae was assayed on microtiter plates or in 2 ml micro-centrifuge tubes, as previously described [68], with concentrated cell suspensions (50–250 million cells per 100 μl) incubated in iron-free Mf medium. ^55^Fe was added to the appropriate concentration in the form of ferrous ascorbate (1:1000) or ferric EDTA (1:5). Iron uptake was stopped after various periods of time, by adding a mixture of strong iron chelators (0.1 mM BPS, 0.1 mM DFOB, and 5 mM EDTA; final concentrations) to the cell suspensions and incubating for 2 min. The cells were then collected with a cell harvester (microtiter plates) or by centrifugation (microcentrifuge tubes), washed three times on the filter with the washing buffer (EDTA/oxalate), and counted in a Wallac 1450 μ Beta TriLux scintillation counter. Cell pigments were bleached with sodium hypochlorite before scintillation counting, to prevent quenching.

### Western blot of Ot-Fea1

A purified polyclonal anti-OtFea1 antibody was raised in rabbit against the peptide CTKYEFPKTRASGNY by GenScript HK Limited. For immunoblot analysis, the cells were lysed in loading buffer (62.4 mM Tris/HCl, 2 % (w/v) sodium dodecyl sulfate, 10 % (w/v) glycerin, 0.01 % (w/v) bromophenol blue, pH 6.8). The lysates were boiled for 5 min, separated (10 μg protein/lane) on an 8 % polyacrylamide gel and electroblotted onto a nitrocellulose membrane. Following the blocking of nonspecific binding sites with 5 % (w/v) milk powder in PBS-T, the membranes were incubated at room temperature with anti-OtFea1 (1:2000 in 5 % (w/v) milk powder in PBS-T). The membranes were washed three times with 5 % (w/v) milk powder in PBS-T (15 min each) and then incubated for 1 h at room temperature with peroxidase-conjugated anti-rabbit-IgY antibody (1:20,000 in 5 % (w/v) milk powder in PBS-T). The membranes were washed three times with 5 % (w/v) milk powder in PBS-T (15 min each), washed three times with PBS-T (15 min each) and developed with Chemiluminescent Peroxidase Substrate-3 (Sigma-Aldrich).

### Chlorophyll *a* determination

Cells were disrupted by sonication with 15 one-second pulses separated by intervals of one second, on ice-water slurry, with an Ultrasonic homogenizer (SONOPULS mini20, Bandelin, equipped with a 2.5 mm-diameter microtip probe). Chlorophyll was extracted by the vigorous vortexing, for one minute, of 100 μl of cell lysate mixed with 900 μl of 90 % acetone, followed by centrifugation at 10,000 × *g* for 10 min. The absorption spectrum of the chlorophyll-containing supernatant was determined directly with a UV-2600 UV-visible light spectrophotometer (Shimadzu), over wavelengths of 600 to 750 nm.

For chlorophyll content determination, we used the equation developed by Porra *et al.* [56] (Chl*a* (μg · ml^−1^) = 12.25 A_663.6_ – 2.55 A_646.6_, where A_663.6_ and A_646.6_, are the absorbance values at wavelengths of 663.6 and 646.6 nm, respectively, minus the absorbance at a wavelength of 750 nm).

### Heme *b* determination

Heme was extracted by a slightly modified version of a published protocol [57]. We prevented sample dilution by performing cell lysis by direct sonication in 350 μl of extraction solution (acetone/HCl 39:1) on ice-water slurry for 20 s. The mixture was vortexed vigorously for 1 min and centrifuged at 10,000 × *g* for 5 min (at room temperature). The heme extract was mixed 1:1 with sample buffer containing 56 mM NH_4_H_2_PO_4_ dissolved in 44 % (vol/vol) HPLC-grade methanol, pH 8.0. Samples were processed within 5 h of preparation, by high-performance liquid chromatography (UltiMate 3000 RSLC, DAD diode array detector, Dionex). HPLC separation began with the loading of 50 μl of sample onto a C18 reverse-phase HPLC column (250 x 4 mm Reprosil 100 C18 5 μm, Watrex Praha) heated to 40 °C. Elution was achieved with a linear gradient from 60/40 % (vol/vol) buffer A/methanol to 100 % methanol at a flow rate of 1.0 ml/min for 25 min. Buffer A consisted of 56 mM NH_4_H_2_PO_4_ dissolved in 44 % (vol/vol) HPLC-grade methanol with the pH adjusted to 3.5 with concentrated orthophosphoric acid. The absorbance of the eluate was assessed at 400 nm. A 1 mg/ml standard solution of heme *b* (hemin, Sigma) was diluted in dimethyl sulfoxide. HPLC was carried out and the data were processed with Chromeleon v7.1.0.898 (Dionex).

### Determination of protein content

Protein levels were determined with the BCA assay, according to the kit manufacturer’s protocol (Sigma-Aldrich). Cells were disrupted by sonication on an ice-water slurry, in 10 mM HEPES pH 7.5 containing protease inhibitors and 1 % Triton X-100. The lysate was centrifuged at 20,000 x g for 15 min at 4 °C and the supernatant was used for protein quantification.

### FPLC analysis

Extracts of cells grown in the presence of ^55^Fe-citrate were separated on a Superdex 200 10/300 GL column (GE Healthcare) at a flow rate of 0.5 lL/min, in 20 mM HEPES and 140 mM NaCl, pH 8.0, with the ÄKTA purifier UPC 10 FPLC system (GE Healthcare). We collected 0.2 ml fractions in a microtiter plate and the radioactivity of each fraction was measured in a scintillation counter (MicoBeta TriLux). The column was calibrated with gel filtration standards (Bio-Rad).

### Native electrophoresis

Cells were disrupted by sonication in the presence of 0.7 % digitonin. Samples were analyzed by blue native PAGE, with the Novex Native PAGE Bis–Tris Gel System (4–16 %), according to the manufacturer’s protocol (Invitrogen). The gels were vacuum-dried and autoradiographed. More specifically (Fig. 5), cells were grown for 5 days in Mf medium containing different amounts of metals. The cells grown in Zn-deficient medium had previously been maintained in Zn-free medium for three months. The cells were harvested, washed once with Mf medium (without Fe, Zn and Cu) and resuspended in the same medium (at 5 x 10^8^ cells/ml) containing 5 μM ^55^Fe(III)-citrate. After 3 h, the cells were harvested and whole-cell extracts were obtained and subjected to native PAGE (25 μg/lane). After autoradiography, iron-containing bands were analyzed by mass spectrometry, as previously described [68].

### Ferrireductase activity

Ferrireductase activity was measured by incubating the cells in the dark for 10–60 min in the corresponding medium containing 0.5 mM Fe(III)-citrate and 2 mM BPS. Cells were harvested from the medium at intervals by centrifugation, and the amount of reduced iron in the medium was calculated.

### Availability of supporting data

The data sets supporting the results of this article are included within the article and its additional files.

## Additional files

Additional file 1: Figure S1. Schematic representation of the experimental design used for analysis of the short-term and long-term adaptive responses of the cells to iron deprivation. In this study, 16 different samples were analyzed with RNAseq technology. They are represented here with gray circles. Transcriptome states were determined in *O. tauri* as a function of three different factors: i) iron deprivation, (−Fe) or control (+Fe), ii) light or dark exposure and iii) short- or long-term adaptive response (condition 1 and condition 2). Experiments comparing conditions 1 and 2 were repeated three times, with three biological replicates for each sample. (PPTX 70 kb) Additional file 2: Figure S2. Bioinformatics protocol used to analyze raw sequencing data (FASTQ files). The quality of read sequences was first checked with the FASTQC program. CDS sequences from *O. tauri* were downloaded from the ORCAE database and used as references for read mapping with the BOWTIE program. BOWTIE was applied with default parameter values. Most of the reads were successfully aligned (>80 %), indicating a high level of sequence quality, and between 5 and 10 million mapped sequences were obtained for each RNAseq sample (data not shown). These sequences were used to estimate gene expression, counting the number of reads mapping to each CDS with the BEDTOOLS program. Expression measurements were thus obtained for more than 80 % of the CDS in *O. tauri* genome, yielding satisfactory sequencing coverage (data not shown). Read counts were normalized with the DESeq program and used to assess the differential expression of genes between conditions (−Fe/+Fe or LIGHT/DARK). Finally, genes were considered differentially expressed if had a log fold-change value greater than 1 or below −1 and a *p*-value (calculated from the biological replicates) below 0.01. (PPTX 432 kb) Additional file 3: Figure S3. Genes identified as differentially expressed between iron conditions. RNAseq results were analyzed with the DESeq program to identify differentially expressed genes, i.e. with significantly different levels of expression between iron nutrition conditions (−Fe and + Fe). (A) Number of genes selected for each time point in each set of experimental conditions. The time points analyzed during the short-term response (condition 1) are shown on the left (3 h – LIGHT, 6 h – LIGHT, 3 h – DARK and 6 h – DARK) and those analyzed during the long-term response (condition 2) are shown on the right (3 h – LIGHT, 9 h – LIGHT, 15 h – DARK, 22 h – DARK). Upregulation is defined as a LogFC > 1 and a *p*-value < 1 % and downregulation is defined as a LogFC < −1 and a *p*-value < 1 %. In total, 128 and 1201 genes were upregulated at one time point at least, in conditions 1 and 2, respectively; 224 and 259 genes were downregulated at one time point at least, in conditions 1 and 2, respectively. (B) Venn diagrams representing the intersections between up- and downregulated genes in conditions 1 and 2. We found that 96 genes were upregulated in both conditions and 57 genes were downregulated in both conditions. A detailed list of the genes is provided in Supplemental Table S1. *Right. Synthetic representation of the genes selected for detailed analysis on the basis of the RNAseq results obtained in this work*. (C) Number of genes according to the number of conditions in which they were identified as differentially expressed (with LogFC > 1 or LogFC < −1 and *p*-value < 1 %). All the genes differentially expressed for at least two conditions were selected for further investigation. Genes differentially expressed in only one set of conditions were selected only in cases of relevant functional information (see main text). A set of 1048 genes was thus finally compiled and described in Supplemental Table S1. (D) Pie chart representing the 19 functional categories manually defined in this work (see main text), with the number of genes assigned to each function. (PPTX 315 kb) Additional file 4: Data Sets 1. Detailed list of the genes whose expression was significantly modified in iron deprivation (−Fe) compared to iron excess (+Fe) condition. (XLS 1381 kb) Additional file 5: Data Sets 2. All information regarding all the genes for which expression was monitored using RNAseq technology. (XLS 7032 kb) Additional file 6: Figure S4. Global comparison of the *O. tauri* and *C. reinhardtii* transcriptional responses to iron limitation. Gene expression data were collected from the article of Urzica *et al.* [72] and orthologous relationships between genes were inferred with the INPARANOID program, using default parameters. (A) Statistics regarding the number of genes for which expression had been quantified, the number of genes with LogFC values > 0.5 or < −0.5 and the percentage of genes with ortholog assignment (based on INPARANOID predictions). (B) Biplots showing the correlation between LogFC values for all orthologous gene pairs (represented as a point on the graph). Pairs with conserved expression patterns in the two species are shown in red (induced) or green (repressed). The associated number of pairs is indicated on the upper right and the bottom left of each graph. The very low proportion of orthologs for which regulation is conserved in the two species reveals fundamental differences in iron metabolism between green algae. (PPTX 443 kb) Additional file 7: Figure S5. Effect of copper (A) and iron (B) on the growth rate of *O. tauri* and *C. reinhardtii,* and iron associated to the cells of both species as a function of iron concentration in the medium (C). A, B: *O. tauri* (red circles) and *C. reinhardtii* (black squares) cells were grown with a gradient of CuSO_4_ (0.01 nM-50 μM) and Fe(III)-citrate (1 nM-10 μM) concentrations as described in Methods. Results are expressed as a % of the maximal growth rate in exponential growth phase. Means ± SD from 4 experiments. C: Intracellular iron content was determined as described in Methods after growing *O. tauri* (red circles) and *C. reinhardtii* (black squares) for 5 days in the presence of 10 nM-10 μM ^55^Fe-ferric citrate (open symbols) or ^55^Fe(III)-EDTA (closed symbols)..Means ± SD from 3 experiments. The maximal growth rates of *O. tauri* and *C. reinhardtii* were reached with 50 nM and >1 μM iron in the medium, respectively (A). Copper also had very different effects on the growth of the two species: the addition of copper to the medium did not improve the growth of *O. tauri* (B), even when the cells were repeatedly used to re-inoculate copper-free medium (*i.e.* in medium with no added copper) over a period of several months. Thus, contaminating copper was sufficient to meet the copper requirements of the cells; when copper was added to the medium, it became toxic at concentrations greater than 1 μM (B). By contrast, the addition of copper to the medium at concentrations of up to 5 μM improved the growth of *C. reinhardtii*, and toxicity was observed only at concentrations exceeding this value (B). These striking differences probably reflect fundamental aspects of the iron-copper connection in the two species, and differences in the response of cells to iron deprivation.The iron content per cell volume was 10 to 100-fold greater in *O. tauri* than in *C. reinhardtii*, suggesting that the iron uptake systems of O. tauri are much more efficient than those of *C. reinhardtii*. (PPTX 272 kb) Additional file 8: Figure S6. Domain organization of ferric reductase and cytochrome b561 homologs in *C. reinhardtii* and *O. tauri.* The ferrireductase homolog (ostta09g01890) was slightly repressed, whereas the two cytochrome b561 homologs (ostta16g00370 and ostta04g02840) were significantly upregulated by iron deprivation in *O. tauri* cells. Domains were identified by HMM-HMM comparison, with the Pfam database. (PPTX 64 kb) Additional file 9: Figure S7. Effect of metals in the medium on iron uptake by *O. tauri.* The cells were maintained in Mf medium without zinc and copper (and 0.1 μM iron) for 3 months. The cells were then harvested and used to inoculate Mf medium with or without supplementation with zinc (0 or 1 μM), iron (1 or 100 nM) and copper (0 or 1 μM). After one week of growth, the cells were harvested and iron uptake kinetics were recorded with 1 μM ^55^Fe(III)-EDTA (1:10). Mean ± SD from 3 experiments. (PPTX 122 kb) Additional file 10: Figure S8. Domain organization of multicopper oxidase and iron permease homologs in *C. reinhardtii* and *O. tauri.* (A) The closest homolog to the C. reinhardtii multicopper oxidase in *O. tauri* has a different organization of multicopper oxidase domains (ostta14g01670) and was not significantly induced by iron deficiency in our study. (B) The only *O. tauri* gene (ostta10g02530) displaying some sequence identity to the Ftr1 iron permease in HMM-HMM analysis had an entirely different domain organization. Domains were identified with the Pfam database and the Phobius transmembrane topology and signal peptide predictor. (PPTX 60 kb) Additional file 11: Figure S9. A. thaliana IRT1 homolog in *O. tauri* contains three His-rich motifs. Pairwise alignment of *A. thaliana* Irt1 and its iron-regulated homolog from *O. tauri* (ostta16g02300). Transmembrane regions were predicted with TMpred software. Blue strips correspond to transmembrane helices. Histidine-rich motifs are framed. The figure was generated with Geneious version 7.1 (Biomatters). (PPTX 615 kb) Additional file 12: Figure S10. The Ot-Fea1 protein contains two Fea1 domains. (A) Multiple alignment of the N- and C-terminal OtFea1 domains and the *C. reinhardtii* Fea1 protein. R/K-E/D-X-X-E motifs are framed. (B) Domain organization of *O. tauri* OtFea1, *P. tricornutum* Isip2a and *C. reinhardtii* Fea1 as revealed by HMM-HMM comparison, with the Pfam database and the Phobius transmembrane topology and signal peptide predictor. (PPTX 781 kb) Additional file 13: Figure S11. Mass spectrometry analysis of the proteins present in the iron-containing band of the native gel shown in Fig. 5. A: LC-MSMS-based Ostreococcus Protein identification from in-gel trypsin digestions of ^55^Fe-labelled proteins (from the iron band shown in Fig. 5). Right and left panels show the results of two independent experiments. Identifications were sorted by descending Mascot scores. Data presented include the Mascot score, the protein sequence coverage (as %), the number of proteins in the identified protein groups, the number of unique peptides, and of peptide search matches, together with the description of the protein in terms of number of aminoacid residues, molecular mass and calculated isoelectric point. B: MSMS-based OtFea sequence coverage. Peptides identified at a 1 % FDR are highlighted in green. C: Representative annotated fragmentation spectrum of an OtFea peptide (Charge: +2, Monoisotopic m/z: 795.88043 Da (−0.27 mmu/-0.34 ppm), MH+: 1590.75359 Da, RT: 27.91 min) and D: corresponding sequence attribution (AQALVADGGVCETGSR, C11-Carbamidomethyl (57.02146 Da)). The peptide was identified with Mascot (v1.30) with an ion Score of 83 and an e-value of 8.2E-008. Fragment match tolerance used for search was 0.02 Da. Fragments used for search were: a; a-H_2_O; a-NH_3_; b; b-H_2_O; b-NH_3_; y; y-H_2_O; y-NH_3_. (PPTX 659 kb) Additional file 14: Figure S12. Comparison of ferritin iron loading in *O. tauri and C. reinhardtii.* Cells of the two species (including the ferritin KO mutant of *O. tauri*, used as a control) were cultured as described in the methods, with different concentrations of Cu (−: no Cu added; +: 0.1 μM (*O. tauri*) or 1 μM (*C. reinhardtii*) CuSO_4_) and Fe (−: 1 nM (*O. tauri*) or 50 nM (*C. reinhardtii*) ferric citrate; +: 1 μM ferric citrate). After 5 days, the cells were harvested, and resuspended in iron-free Mf medium (*O. tauri*) or iron-free TAP medium (*C. reinhardtii*) supplemented with 5 μM ^55^Fe(III)-citrate. The cells were incubated for 3 h and then harvested by centrifugation. Whole-cell extracts were obtained and subjected to native PAGE (25 μg/lane), as described in the methods. A comparison of autoradiographs of dried gels shows that *O. tauri* ferritin was maximally loaded with iron when the cells had previously been grown in iron-rich conditions, whereas the main ferritin of *C. reinhardtii* was maximally loaded with iron when the cells were grown in iron-deficient conditions. The addition of Cu to the medium resulted in some intracellular iron redistribution in *O. tauri* (although Cu did not affect iron uptake): a band that we previously identified as a probable nitrate reductase (Botebol et al.) was more intense in the presence of copper. We are currently characterizing this band further. (PPTX 3065 kb) Additional file 15: Figure S13. Gel filtration elution profile (proteins and iron) of a whole-cell extract of *O. tauri cells. O. tauri* cells were grown for one week with 0.1 μM ^55^Fe(III) citrate as an iron source. The cells were disrupted by sonication in the presence of 0.5 % digitonin, and the soluble fraction was subjected to gel filtration (FPLC) as described in the methods. The black curve shows the elution profile of the proteins, and the red curve shows the elution profile of ^55^Fe. Iron was associated with three main fractions: i) a very high-MW fraction, corresponding to the high-MW complexes of the photosystems and respiratory chain, ii) a high-MW fraction corresponding to ferritin, and other large iron complexes, such as nitrate reductase, and iii) a very low-MW fraction corresponding to the unknown iron pool. This last fraction accounted for most of the intracellular iron, probably stored in the form of polyphosphate complexes. (PPTX 157 kb)
