# Supplementary material for: Ostreococcus tauri is a new model green alga for studying iron metabolism in eukaryotic phytoplankton
Source: BMC Genomics. 2016 May 3;17:319. doi: 10.1186/s12864-016-2666-6 (PMC4855317; doi:10.1186/s12864-016-2666-6)
Supplement: Additional file 1: Figure S1. — Schematic representation of the experimental design used for analysis of the short-term and long-term adaptive responses of the cells to iron deprivation. In this study, 16 different samples were analyzed with RNAseq technology. They are represented here with gray circles. Transcriptome states were determined in O. tauri as a function of three different factors: i) iron deprivation, (−Fe) or control (+Fe), ii) light or dark exposure and iii) short- or long-term adaptive response (condition 1 and condition 2). Experiments comparing conditions 1 and 2 were repeated three times, with three biological replicates for each sample. (PPTX 70 kb) [file 12864_2016_2666_MOESM1_ESM.pptx]

## Slide 1
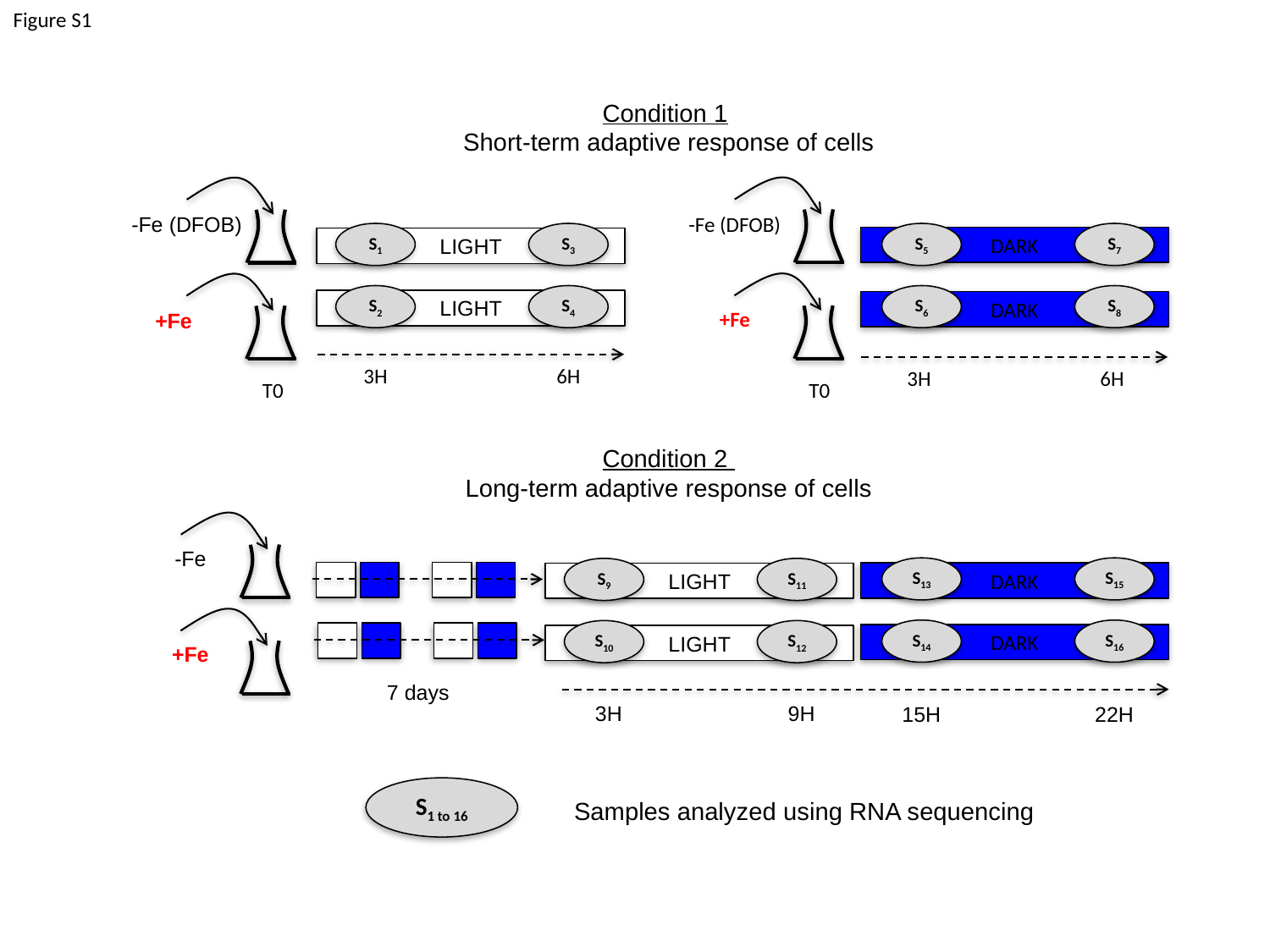

Figure S1
Condition 1
Short-term adaptive response of cells
-Fe (DFOB)
S1
S3
S2
S4
S5
S7
S6
S8
DARK
DARK
LIGHT
LIGHT
+Fe
3H
6H
3H
6H
T0
T0
Condition 2
Long-term adaptive response of cells
S13
S15
S14
S16
S9
S11
S10
S12
DARK
DARK
LIGHT
LIGHT
7 days
3H
9H
15H
22H
S1 to 16
Samples analyzed using RNA sequencing
-Fe (DFOB)
-Fe
+Fe
+Fe
