# Supplementary material for: Ostreococcus tauri is a new model green alga for studying iron metabolism in eukaryotic phytoplankton
Source: BMC Genomics. 2016 May 3;17:319. doi: 10.1186/s12864-016-2666-6 (PMC4855317; doi:10.1186/s12864-016-2666-6)
Supplement: Additional file 2: Figure S2. — Bioinformatics protocol used to analyze raw sequencing data (FASTQ files). The quality of read sequences was first checked with the FASTQC program. CDS sequences from O. tauri were downloaded from the ORCAE database and used as references for read mapping with the BOWTIE program. BOWTIE was applied with default parameter values. Most of the reads were successfully aligned (>80 %), indicating a high level of sequence quality, and between 5 and 10 million mapped sequences were obtained for each RNAseq sample (data not shown). These sequences were used to estimate gene expression, counting the number of reads mapping to each CDS with the BEDTOOLS program. Expression measurements were thus obtained for more than 80 % of the CDS in O. tauri genome, yielding satisfactory sequencing coverage (data not shown). Read counts were normalized with the DESeq program and used to assess the differential expression of genes between conditions (−Fe/+Fe or LIGHT/DARK). Finally, genes were considered differentially expressed if had a log fold-change value greater than 1 or below −1 and a p-value (calculated from the biological replicates) below 0.01. (PPTX 432 kb) [file 12864_2016_2666_MOESM2_ESM.pptx]

## Slide 1
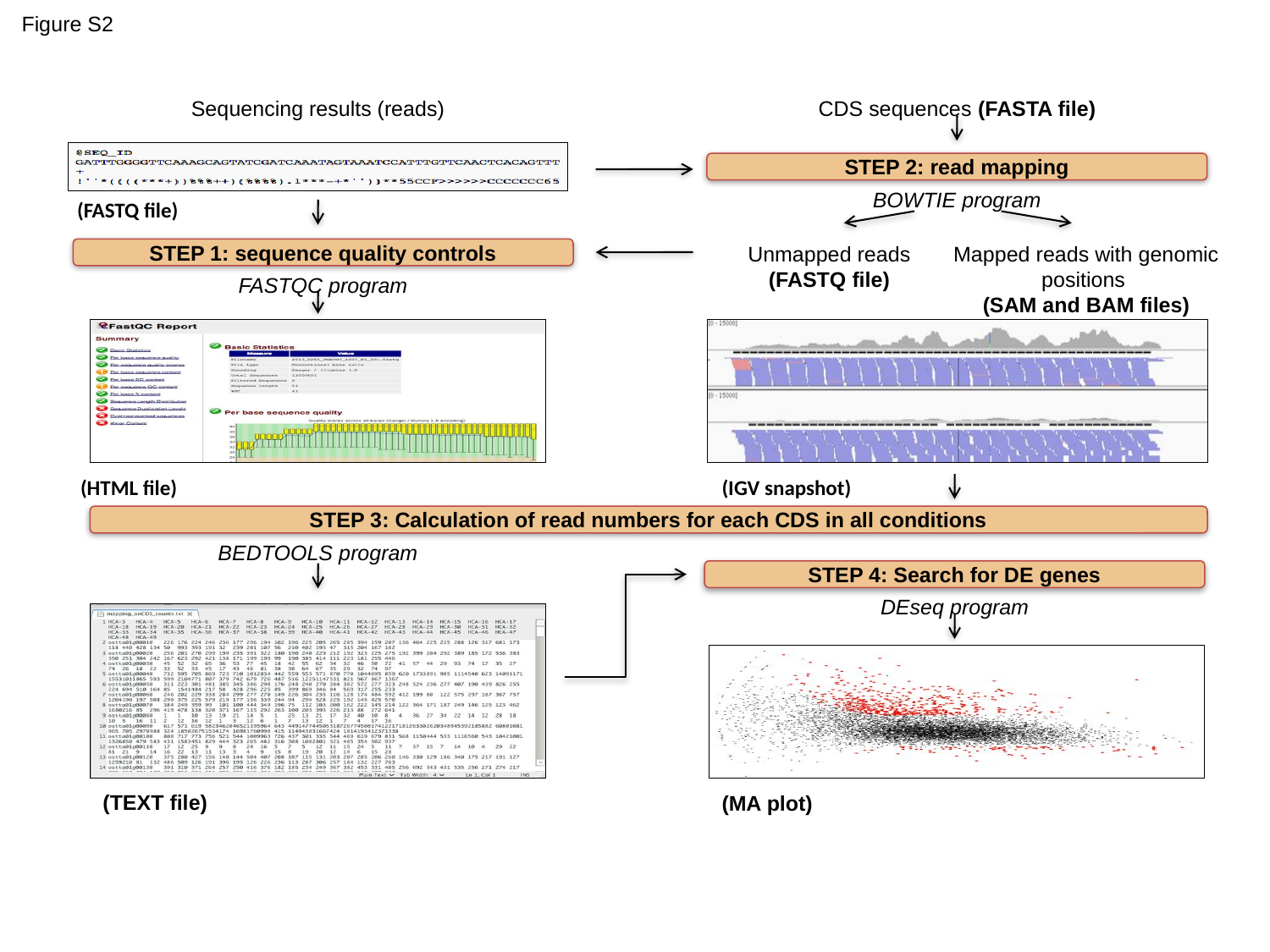

Figure S2
Sequencing results (reads)
CDS sequences (FASTA file)
STEP 2: read mapping
BOWTIE program
(FASTQ file)
Unmapped reads
(FASTQ file)
Mapped reads with genomic positions
(SAM and BAM files)
STEP 1: sequence quality controls
FASTQC program
(HTML file)
(IGV snapshot)
STEP 3: Calculation of read numbers for each CDS in all conditions
BEDTOOLS program
STEP 4: Search for DE genes
DEseq program
(TEXT file)
(MA plot)
