# Supplementary figures and images for: Ostreococcus tauri is a new model green alga for studying iron metabolism in eukaryotic phytoplankton
Source: BMC Genomics. 2016 May 3;17:319. doi: 10.1186/s12864-016-2666-6 (PMC4855317; doi:10.1186/s12864-016-2666-6)

## Slide 1
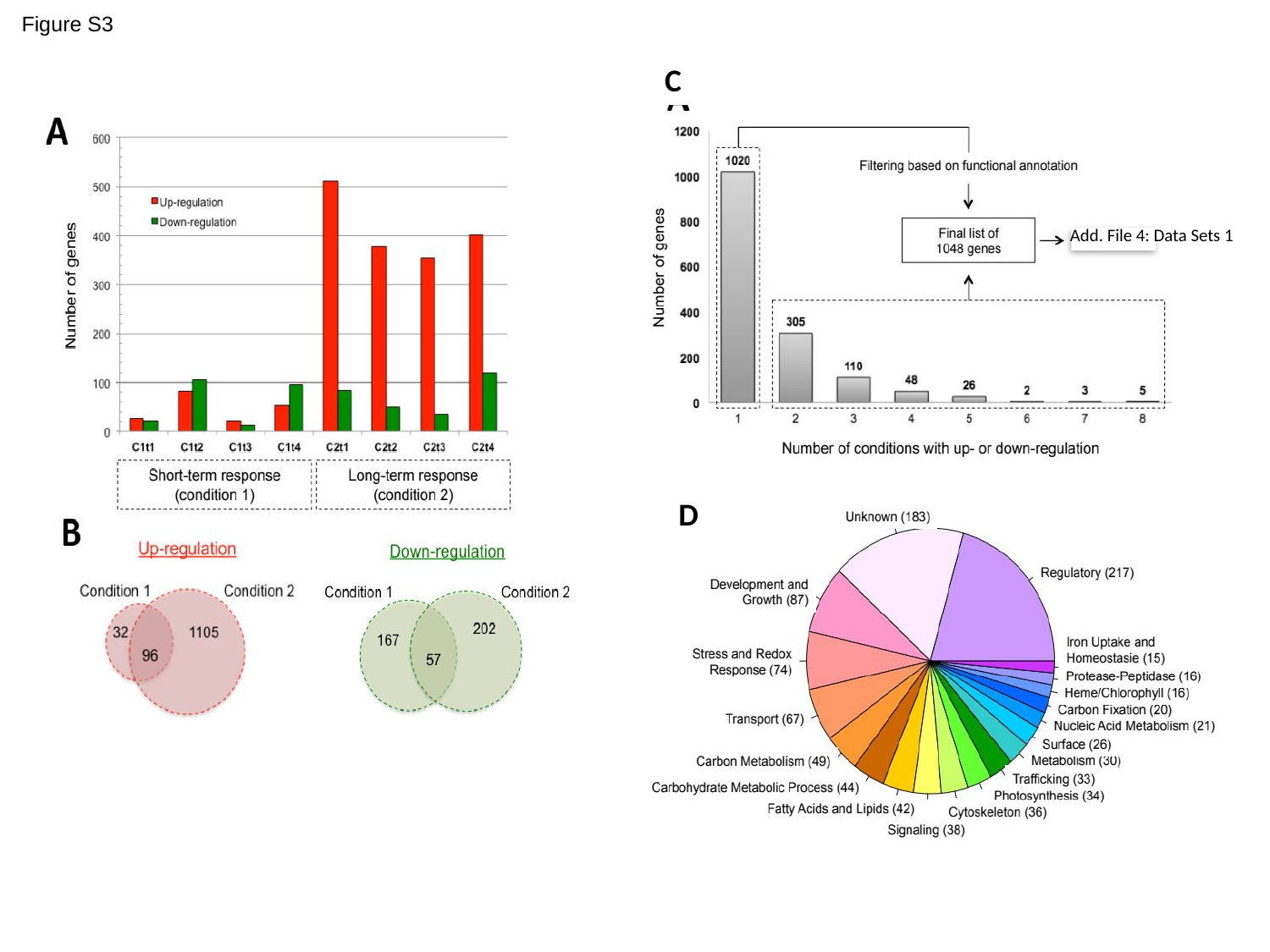

Figure S3
C
D
Add. File 4: Data Sets 1

Supplement: Additional file 3: Figure S3. — Genes identified as differentially expressed between iron conditions. RNAseq results were analyzed with the DESeq program to identify differentially expressed genes, i.e. with significantly different levels of expression between iron nutrition conditions (−Fe and + Fe). (A) Number of genes selected for each time point in each set of experimental conditions. The time points analyzed during the short-term response (condition 1) are shown on the left (3 h – LIGHT, 6 h – LIGHT, 3 h – DARK and 6 h – DARK) and those analyzed during the long-term response (condition 2) are shown on the right (3 h – LIGHT, 9 h – LIGHT, 15 h – DARK, 22 h – DARK). Upregulation is defined as a LogFC > 1 and a p-value < 1 % and downregulation is defined as a LogFC < −1 and a p-value < 1 %. In total, 128 and 1201 genes were upregulated at one time point at least, in conditions 1 and 2, respectively; 224 and 259 genes were downregulated at one time point at least, in conditions 1 and 2, respectively. (B) Venn diagrams representing the intersections between up- and downregulated genes in conditions 1 and 2. We found that 96 genes were upregulated in both conditions and 57 genes were downregulated in both conditions. A detailed list of the genes is provided in Supplemental Table S1. Right. Synthetic representation of the genes selected for detailed analysis on the basis of the RNAseq results obtained in this work. (C) Number of genes according to the number of conditions in which they were identified as differentially expressed (with LogFC > 1 or LogFC < −1 and p-value < 1 %). All the genes differentially expressed for at least two conditions were selected for further investigation. Genes differentially expressed in only one set of conditions were selected only in cases of relevant functional information (see main text). A set of 1048 genes was thus finally compiled and described in Supplemental Table S1. (D) Pie chart representing the 19 functional categories manually defined in this work (see mai [file 12864_2016_2666_MOESM3_ESM.pptx]

## Slide 1
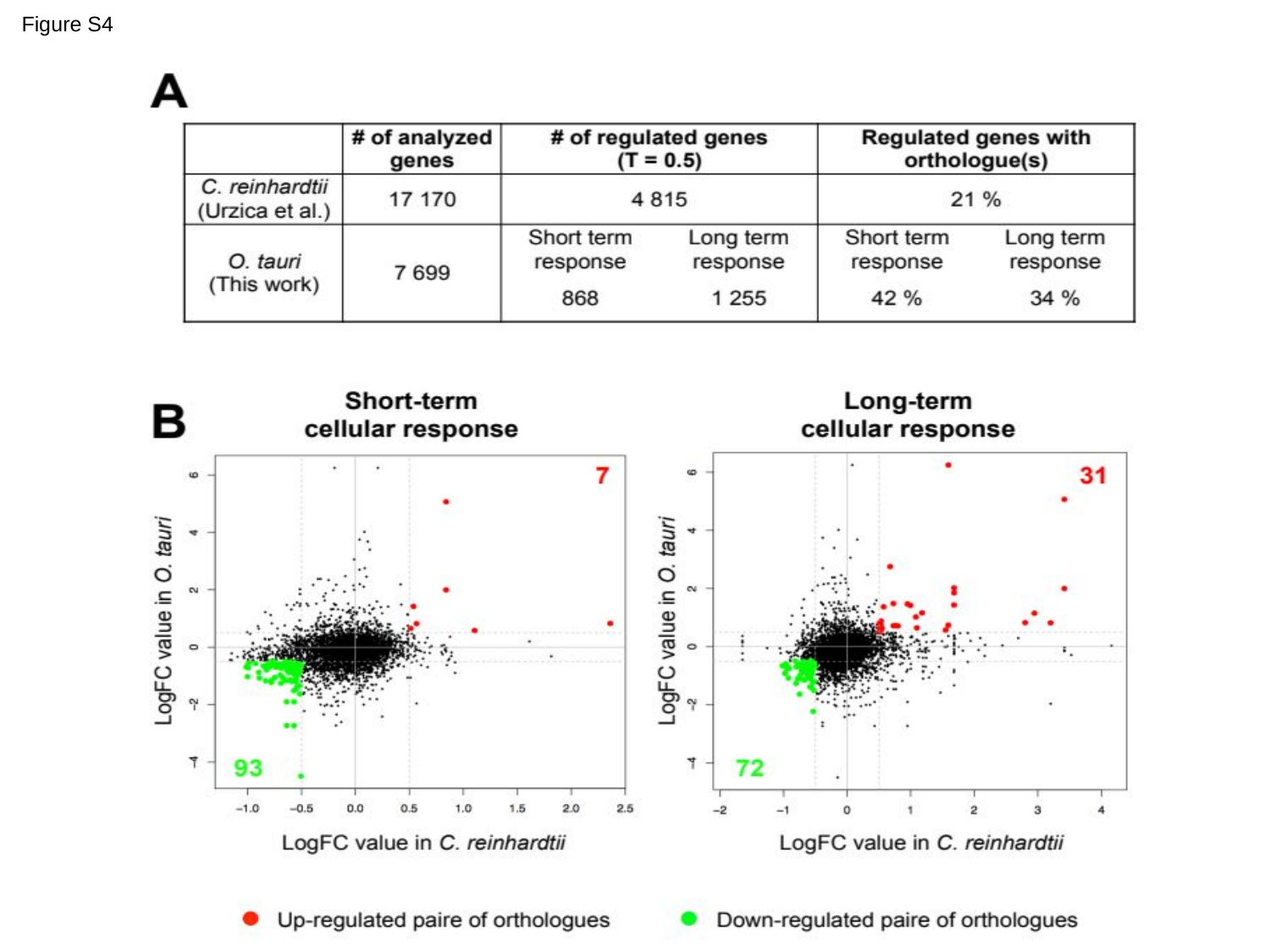

Figure S4

Supplement: Additional file 6: Figure S4. — Global comparison of the O. tauri and C. reinhardtii transcriptional responses to iron limitation. Gene expression data were collected from the article of Urzica et al. [72] and orthologous relationships between genes were inferred with the INPARANOID program, using default parameters. (A) Statistics regarding the number of genes for which expression had been quantified, the number of genes with LogFC values > 0.5 or < −0.5 and the percentage of genes with ortholog assignment (based on INPARANOID predictions). (B) Biplots showing the correlation between LogFC values for all orthologous gene pairs (represented as a point on the graph). Pairs with conserved expression patterns in the two species are shown in red (induced) or green (repressed). The associated number of pairs is indicated on the upper right and the bottom left of each graph. The very low proportion of orthologs for which regulation is conserved in the two species reveals fundamental differences in iron metabolism between green algae. (PPTX 443 kb) [file 12864_2016_2666_MOESM6_ESM.pptx]

## Slide 1
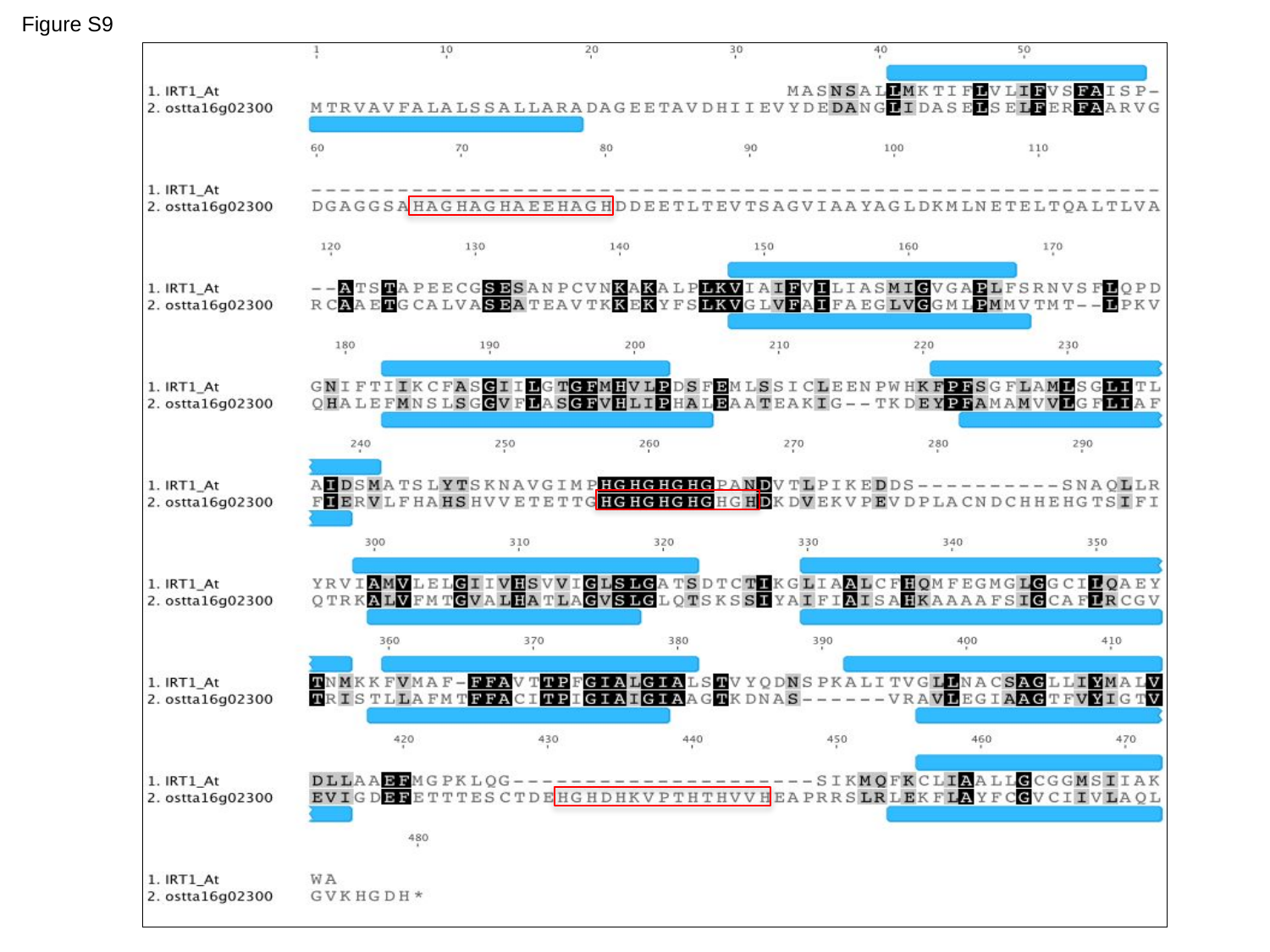

Figure S9

Supplement: Additional file 11: Figure S9. — A. thaliana IRT1 homolog in O. tauri contains three His-rich motifs. Pairwise alignment of A. thaliana Irt1 and its iron-regulated homolog from O. tauri (ostta16g02300). Transmembrane regions were predicted with TMpred software. Blue strips correspond to transmembrane helices. Histidine-rich motifs are framed. The figure was generated with Geneious version 7.1 (Biomatters). (PPTX 615 kb) [file 12864_2016_2666_MOESM11_ESM.pptx]

## Slide 1
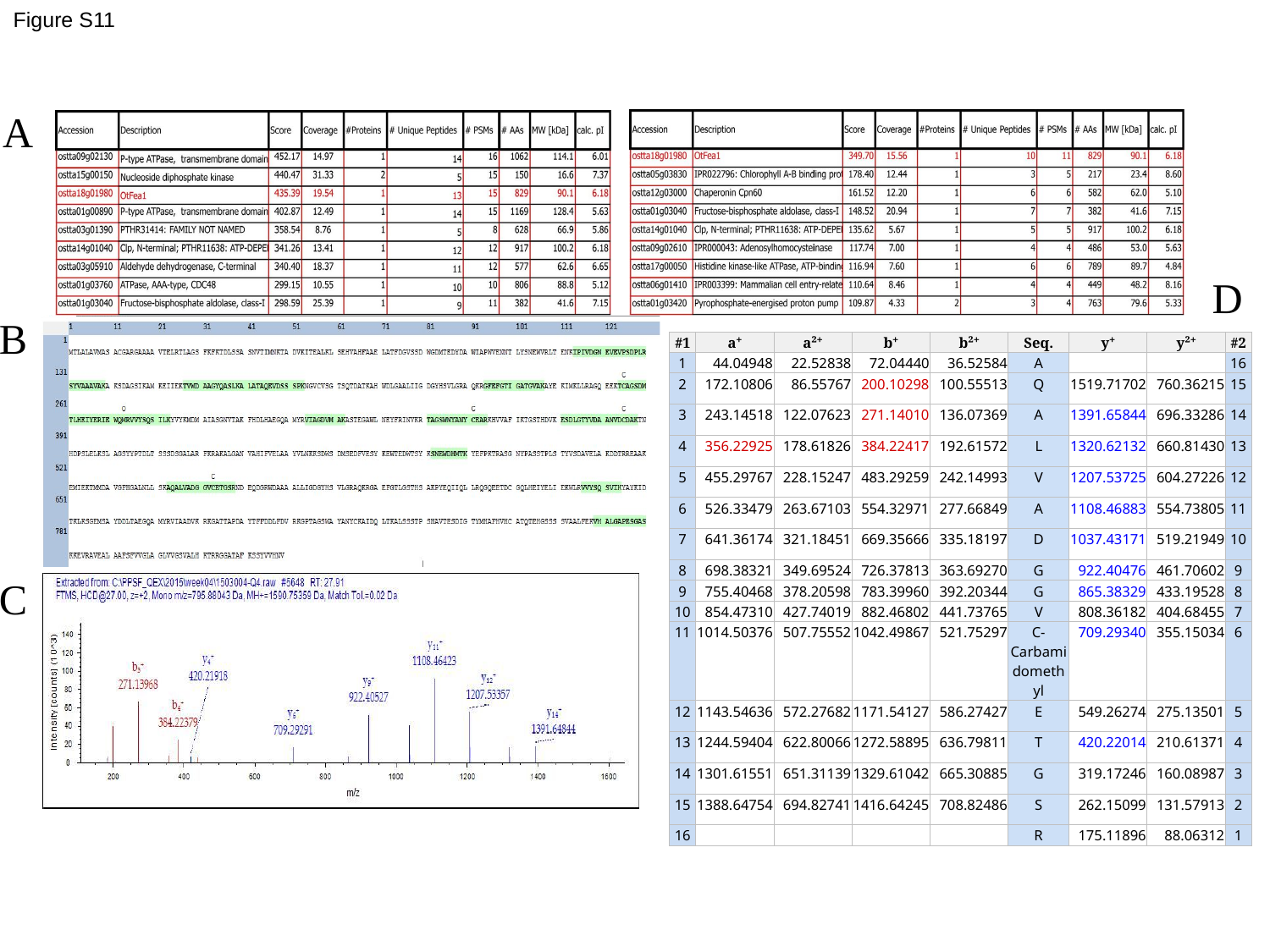

Figure S11
A
D
B
| #1 | a⁺ | a²⁺ | b⁺ | b²⁺ | Seq. | y⁺ | y²⁺ | #2 |
| --- | --- | --- | --- | --- | --- | --- | --- | --- |
| 1 | 44.04948 | 22.52838 | 72.04440 | 36.52584 | A | | | 16 |
| 2 | 172.10806 | 86.55767 | 200.10298 | 100.55513 | Q | 1519.71702 | 760.36215 | 15 |
| 3 | 243.14518 | 122.07623 | 271.14010 | 136.07369 | A | 1391.65844 | 696.33286 | 14 |
| 4 | 356.22925 | 178.61826 | 384.22417 | 192.61572 | L | 1320.62132 | 660.81430 | 13 |
| 5 | 455.29767 | 228.15247 | 483.29259 | 242.14993 | V | 1207.53725 | 604.27226 | 12 |
| 6 | 526.33479 | 263.67103 | 554.32971 | 277.66849 | A | 1108.46883 | 554.73805 | 11 |
| 7 | 641.36174 | 321.18451 | 669.35666 | 335.18197 | D | 1037.43171 | 519.21949 | 10 |
| 8 | 698.38321 | 349.69524 | 726.37813 | 363.69270 | G | 922.40476 | 461.70602 | 9 |
| 9 | 755.40468 | 378.20598 | 783.39960 | 392.20344 | G | 865.38329 | 433.19528 | 8 |
| 10 | 854.47310 | 427.74019 | 882.46802 | 441.73765 | V | 808.36182 | 404.68455 | 7 |
| 11 | 1014.50376 | 507.75552 | 1042.49867 | 521.75297 | C-Carbamidomethyl | 709.29340 | 355.15034 | 6 |
| 12 | 1143.54636 | 572.27682 | 1171.54127 | 586.27427 | E | 549.26274 | 275.13501 | 5 |
| 13 | 1244.59404 | 622.80066 | 1272.58895 | 636.79811 | T | 420.22014 | 210.61371 | 4 |
| 14 | 1301.61551 | 651.31139 | 1329.61042 | 665.30885 | G | 319.17246 | 160.08987 | 3 |
| 15 | 1388.64754 | 694.82741 | 1416.64245 | 708.82486 | S | 262.15099 | 131.57913 | 2 |
| 16 | | | | | R | 175.11896 | 88.06312 | 1 |
C

Supplement: Additional file 13: Figure S11. — Mass spectrometry analysis of the proteins present in the iron-containing band of the native gel shown in Fig. 5. A: LC-MSMS-based Ostreococcus Protein identification from in-gel trypsin digestions of 55Fe-labelled proteins (from the iron band shown in Fig. 5). Right and left panels show the results of two independent experiments. Identifications were sorted by descending Mascot scores. Data presented include the Mascot score, the protein sequence coverage (as %), the number of proteins in the identified protein groups, the number of unique peptides, and of peptide search matches, together with the description of the protein in terms of number of aminoacid residues, molecular mass and calculated isoelectric point. B: MSMS-based OtFea sequence coverage. Peptides identified at a 1 % FDR are highlighted in green. C: Representative annotated fragmentation spectrum of an OtFea peptide (Charge: +2, Monoisotopic m/z: 795.88043 Da (−0.27 mmu/-0.34 ppm), MH+: 1590.75359 Da, RT: 27.91 min) and D: corresponding sequence attribution (AQALVADGGVCETGSR, C11-Carbamidomethyl (57.02146 Da)). The peptide was identified with Mascot (v1.30) with an ion Score of 83 and an e-value of 8.2E-008. Fragment match tolerance used for search was 0.02 Da. Fragments used for search were: a; a-H2O; a-NH3; b; b-H2O; b-NH3; y; y-H2O; y-NH3. (PPTX 659 kb) [file 12864_2016_2666_MOESM13_ESM.pptx]

## Slide 1
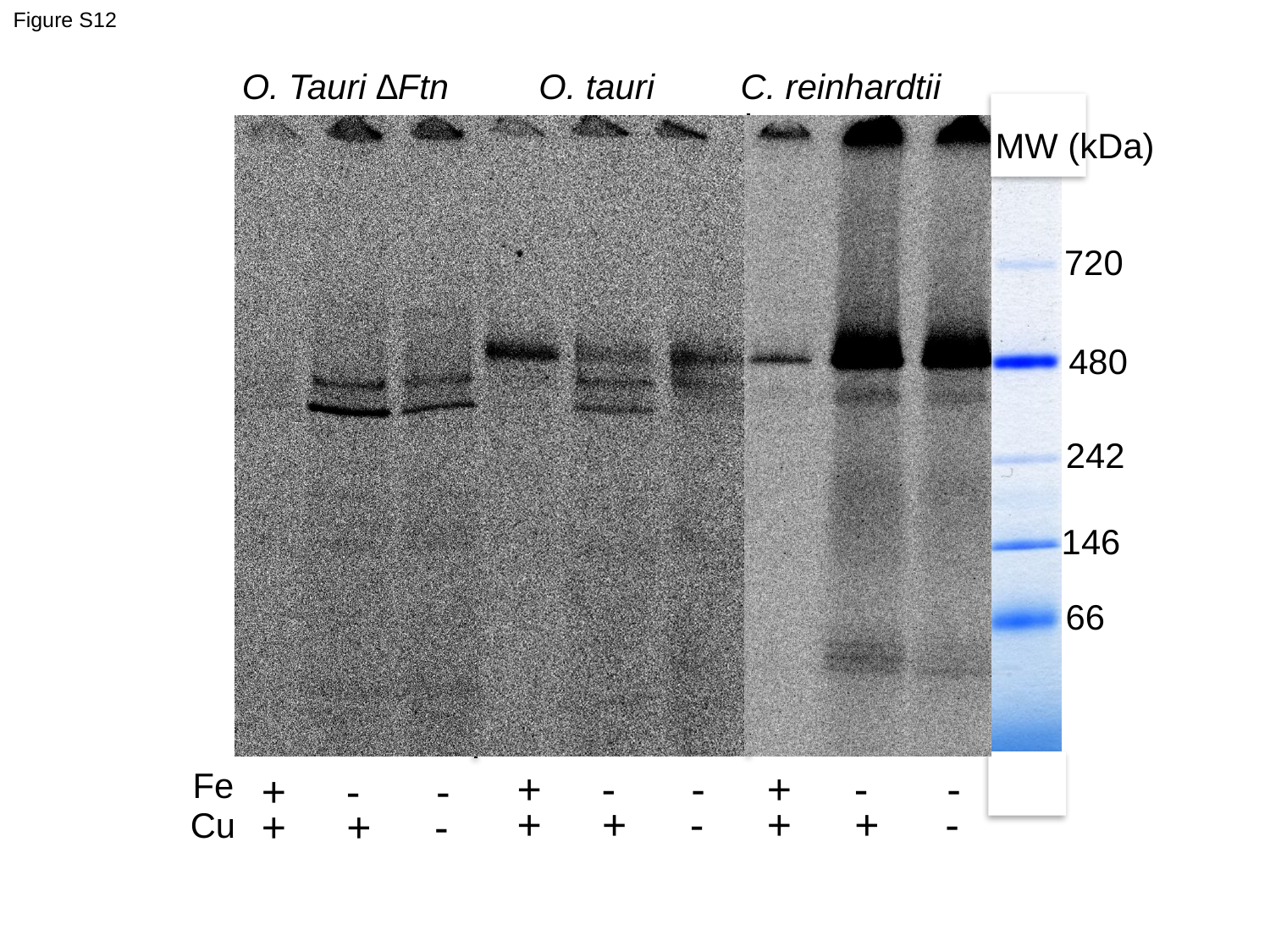

Figure S12
O. Tauri ∆Ftn
O. tauri
C. reinhardtii
MW (kDa)
720
480
*
242
146
66
+
-
-
+
+
-
+
-
-
Fe
+
-
-
+
+
-
+
+
-
Cu

Supplement: Additional file 14: Figure S12. — Comparison of ferritin iron loading in O. tauri and C. reinhardtii. Cells of the two species (including the ferritin KO mutant of O. tauri, used as a control) were cultured as described in the methods, with different concentrations of Cu (−: no Cu added; +: 0.1 μM (O. tauri) or 1 μM (C. reinhardtii) CuSO4) and Fe (−: 1 nM (O. tauri) or 50 nM (C. reinhardtii) ferric citrate; +: 1 μM ferric citrate). After 5 days, the cells were harvested, and resuspended in iron-free Mf medium (O. tauri) or iron-free TAP medium (C. reinhardtii) supplemented with 5 μM 55Fe(III)-citrate. The cells were incubated for 3 h and then harvested by centrifugation. Whole-cell extracts were obtained and subjected to native PAGE (25 μg/lane), as described in the methods. A comparison of autoradiographs of dried gels shows that O. tauri ferritin was maximally loaded with iron when the cells had previously been grown in iron-rich conditions, whereas the main ferritin of C. reinhardtii was maximally loaded with iron when the cells were grown in iron-deficient conditions. The addition of Cu to the medium resulted in some intracellular iron redistribution in O. tauri (although Cu did not affect iron uptake): a band that we previously identified as a probable nitrate reductase (Botebol et al.) was more intense in the presence of copper. We are currently characterizing this band further. (PPTX 3065 kb) [file 12864_2016_2666_MOESM14_ESM.pptx]

## Slide 1
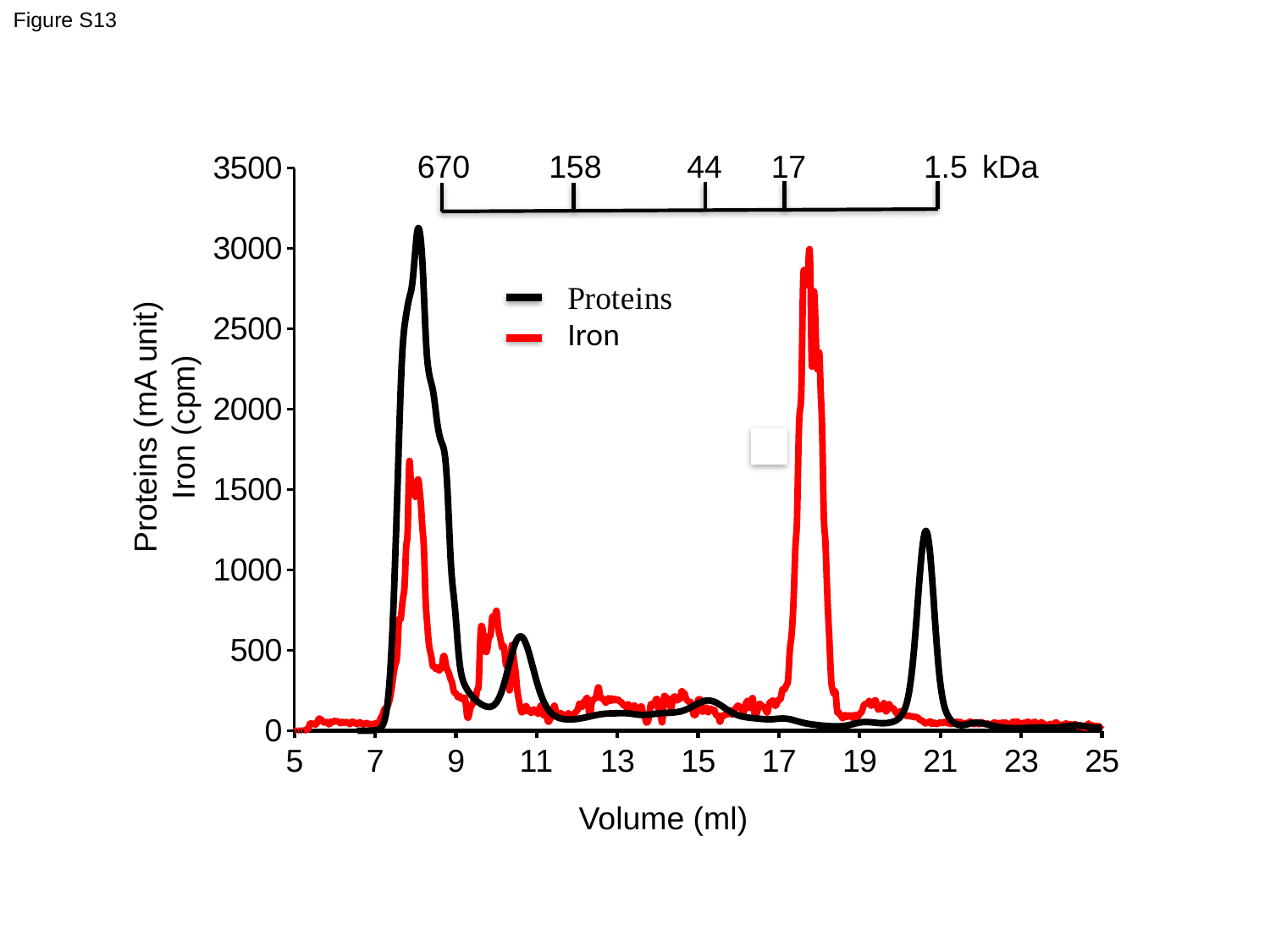

Figure S13
### Chart
| Category | Valeur Y 1 | Valeur Y 2 |
|---|---|---|670
158
44
17
1.5
kDa
Proteins (mA unit)
Iron (cpm)
Volume (ml)

Supplement: Additional file 15: Figure S13. — Gel filtration elution profile (proteins and iron) of a whole-cell extract of O. tauri cells. O. tauri cells were grown for one week with 0.1 μM 55Fe(III) citrate as an iron source. The cells were disrupted by sonication in the presence of 0.5 % digitonin, and the soluble fraction was subjected to gel filtration (FPLC) as described in the methods. The black curve shows the elution profile of the proteins, and the red curve shows the elution profile of 55Fe. Iron was associated with three main fractions: i) a very high-MW fraction, corresponding to the high-MW complexes of the photosystems and respiratory chain, ii) a high-MW fraction corresponding to ferritin, and other large iron complexes, such as nitrate reductase, and iii) a very low-MW fraction corresponding to the unknown iron pool. This last fraction accounted for most of the intracellular iron, probably stored in the form of polyphosphate complexes. (PPTX 157 kb) [file 12864_2016_2666_MOESM15_ESM.pptx]
