# Supplementary material for: Ostreococcus tauri is a new model green alga for studying iron metabolism in eukaryotic phytoplankton
Source: BMC Genomics. 2016 May 3;17:319. doi: 10.1186/s12864-016-2666-6 (PMC4855317; doi:10.1186/s12864-016-2666-6)
Supplement: Additional file 7: Figure S5. — Effect of copper (A) and iron (B) on the growth rate of O. tauri and C. reinhardtii, and iron associated to the cells of both species as a function of iron concentration in the medium (C). A, B: O. tauri (red circles) and C. reinhardtii (black squares) cells were grown with a gradient of CuSO4 (0.01 nM-50 μM) and Fe(III)-citrate (1 nM-10 μM) concentrations as described in Methods. Results are expressed as a % of the maximal growth rate in exponential growth phase. Means ± SD from 4 experiments. C: Intracellular iron content was determined as described in Methods after growing O. tauri (red circles) and C. reinhardtii (black squares) for 5 days in the presence of 10 nM-10 μM 55Fe-ferric citrate (open symbols) or 55Fe(III)-EDTA (closed symbols)..Means ± SD from 3 experiments. The maximal growth rates of O. tauri and C. reinhardtii were reached with 50 nM and >1 μM iron in the medium, respectively (A). Copper also had very different effects on the growth of the two species: the addition of copper to the medium did not improve the growth of O. tauri (B), even when the cells were repeatedly used to re-inoculate copper-free medium (i.e. in medium with no added copper) over a period of several months. Thus, contaminating copper was sufficient to meet the copper requirements of the cells; when copper was added to the medium, it became toxic at concentrations greater than 1 μM (B). By contrast, the addition of copper to the medium at concentrations of up to 5 μM improved the growth of C. reinhardtii, and toxicity was observed only at concentrations exceeding this value (B). These striking differences probably reflect fundamental aspects of the iron-copper connection in the two species, and differences in the response of cells to iron deprivation.The iron content per cell volume was 10 to 100-fold greater in O. tauri than in C. reinhardtii, suggesting that the iron uptake systems of O. tauri are much more efficient than those of C. reinhardtii. (PPTX 272 kb) [file 12864_2016_2666_MOESM7_ESM.pptx]

## Slide 1
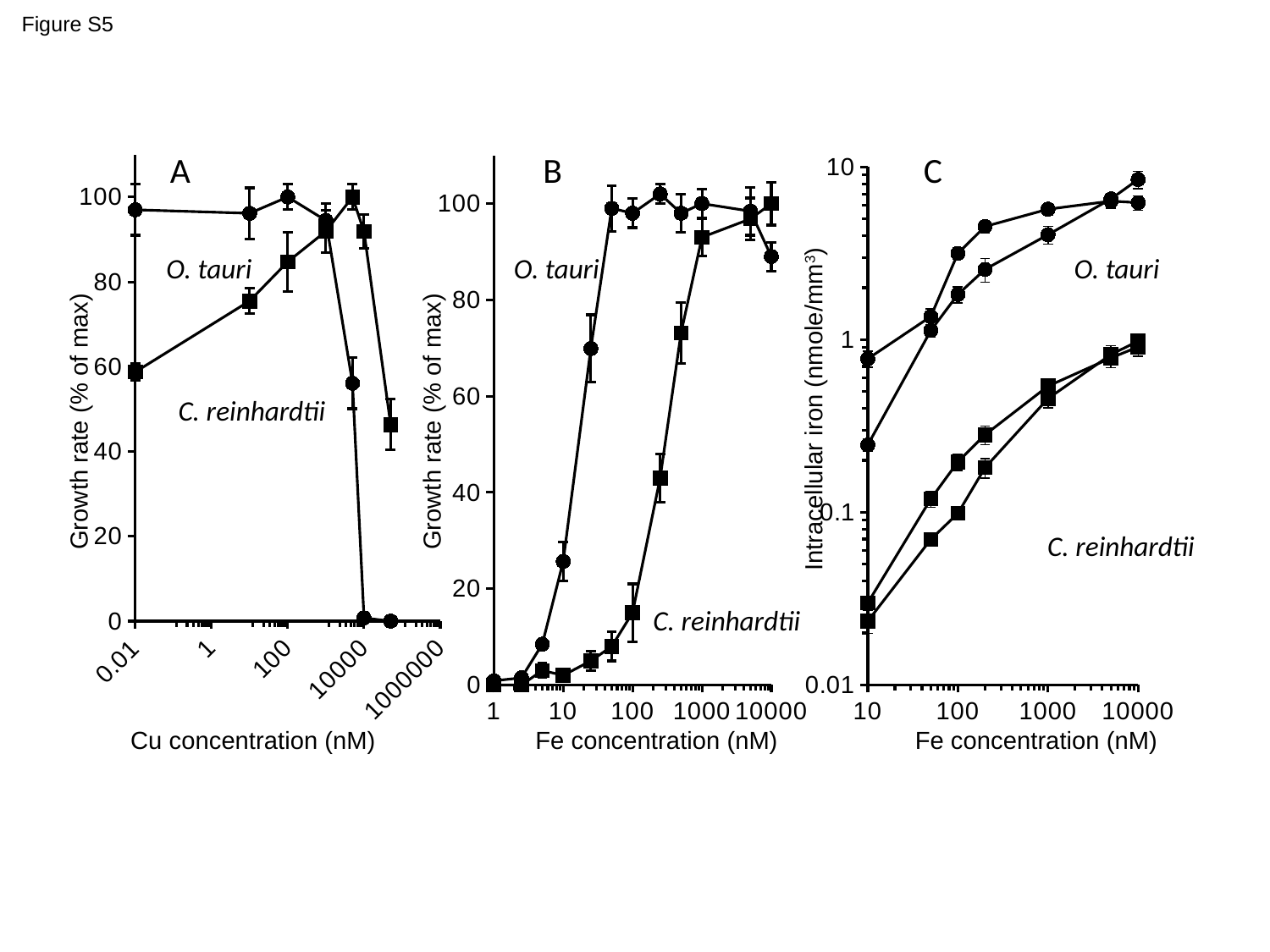

Figure S5
A
B
C
### Chart
| Category | Valeur Y 1 | Valeur Y 2 | Valeur Y 3 | Valeur Y 4 |
|---|---|---|---|---|
### Chart
| Category | Valeur Y 1 | Valeur Y 2 |
|---|---|---|
### Chart
| Category | Valeur Y 1 | Valeur Y 2 |
|---|---|---|O. tauri
O. tauri
O. tauri
C. reinhardtii
Intracellular iron (nmole/mm3)
Growth rate (% of max)
Growth rate (% of max)
C. reinhardtii
C. reinhardtii
Cu concentration (nM)
Fe concentration (nM)
Fe concentration (nM)
