# Supplementary material for: Ostreococcus tauri is a new model green alga for studying iron metabolism in eukaryotic phytoplankton
Source: BMC Genomics. 2016 May 3;17:319. doi: 10.1186/s12864-016-2666-6 (PMC4855317; doi:10.1186/s12864-016-2666-6)
Supplement: Additional file 8: Figure S6. — Domain organization of ferric reductase and cytochrome b561 homologs in C. reinhardtii and O. tauri. The ferrireductase homolog (ostta09g01890) was slightly repressed, whereas the two cytochrome b561 homologs (ostta16g00370 and ostta04g02840) were significantly upregulated by iron deprivation in O. tauri cells. Domains were identified by HMM-HMM comparison, with the Pfam database. (PPTX 64 kb) [file 12864_2016_2666_MOESM8_ESM.pptx]

## Slide 1
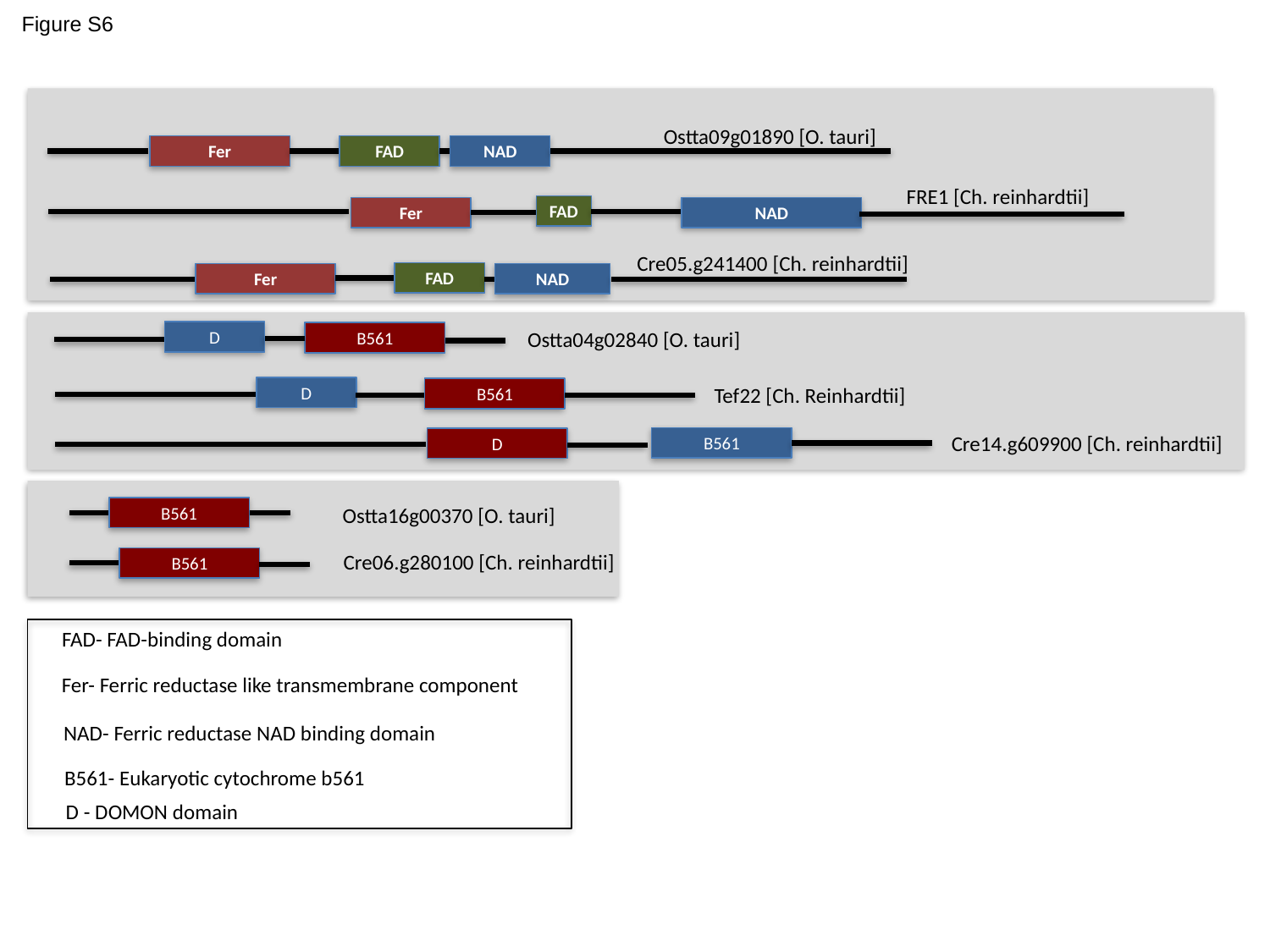

Figure S6
Ostta09g01890 [O. tauri]
Fer
FAD
NAD
FRE1 [Ch. reinhardtii]
FAD
Fer
NAD
Cre05.g241400 [Ch. reinhardtii]
FAD
Fer
NAD
Ostta04g02840 [O. tauri]
D
B561
Tef22 [Ch. Reinhardtii]
D
B561
Cre14.g609900 [Ch. reinhardtii]
B561
D
Ostta16g00370 [O. tauri]
B561
Cre06.g280100 [Ch. reinhardtii]
B561
FAD- FAD-binding domain
Fer- Ferric reductase like transmembrane component
NAD- Ferric reductase NAD binding domain
B561- Eukaryotic cytochrome b561
D - DOMON domain
