# Supplementary material for: Ostreococcus tauri is a new model green alga for studying iron metabolism in eukaryotic phytoplankton
Source: BMC Genomics. 2016 May 3;17:319. doi: 10.1186/s12864-016-2666-6 (PMC4855317; doi:10.1186/s12864-016-2666-6)
Supplement: Additional file 9: Figure S7. — Effect of metals in the medium on iron uptake by O. tauri. The cells were maintained in Mf medium without zinc and copper (and 0.1 μM iron) for 3 months. The cells were then harvested and used to inoculate Mf medium with or without supplementation with zinc (0 or 1 μM), iron (1 or 100 nM) and copper (0 or 1 μM). After one week of growth, the cells were harvested and iron uptake kinetics were recorded with 1 μM 55Fe(III)-EDTA (1:10). Mean ± SD from 3 experiments. (PPTX 122 kb) [file 12864_2016_2666_MOESM9_ESM.pptx]

## Slide 1
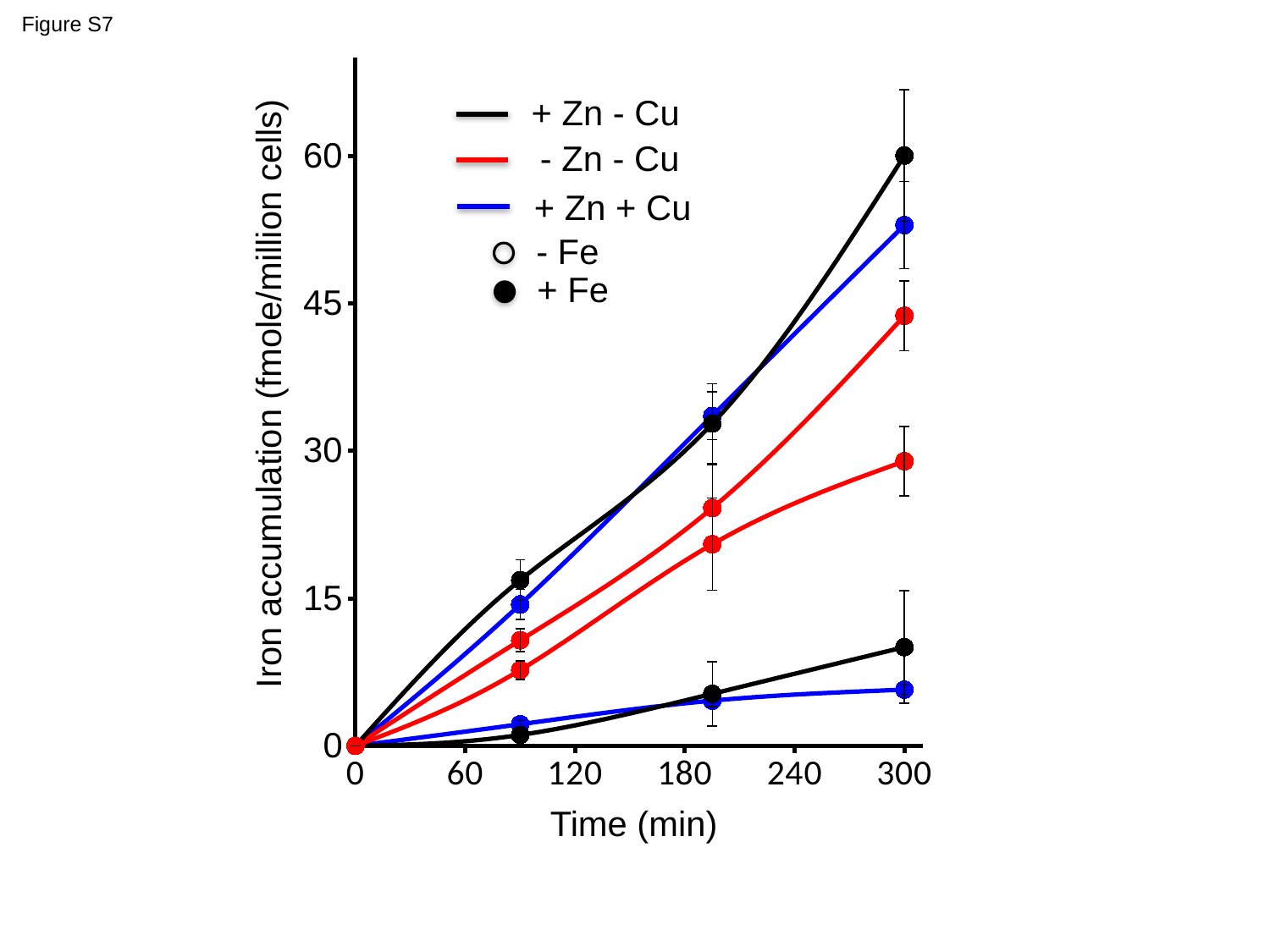

Figure S7
### Chart
| Category | Valeur Y 1 | Valeur Y 2 | Valeur Y 3 | Valeur Y 4 | Valeur Y 5 | Valeur Y 6 |
|---|---|---|---|---|---|---|+ Zn - Cu
- Zn - Cu
+ Zn + Cu
- Fe
+ Fe
Iron accumulation (fmole/million cells)
Time (min)
