# Supplementary material for: Ostreococcus tauri is a new model green alga for studying iron metabolism in eukaryotic phytoplankton
Source: BMC Genomics. 2016 May 3;17:319. doi: 10.1186/s12864-016-2666-6 (PMC4855317; doi:10.1186/s12864-016-2666-6)
Supplement: Additional file 10: Figure S8. — Domain organization of multicopper oxidase and iron permease homologs in C. reinhardtii and O. tauri. (A) The closest homolog to the C. reinhardtii multicopper oxidase in O. tauri has a different organization of multicopper oxidase domains (ostta14g01670) and was not significantly induced by iron deficiency in our study. (B) The only O. tauri gene (ostta10g02530) displaying some sequence identity to the Ftr1 iron permease in HMM-HMM analysis had an entirely different domain organization. Domains were identified with the Pfam database and the Phobius transmembrane topology and signal peptide predictor. (PPTX 60 kb) [file 12864_2016_2666_MOESM10_ESM.pptx]

## Slide 1
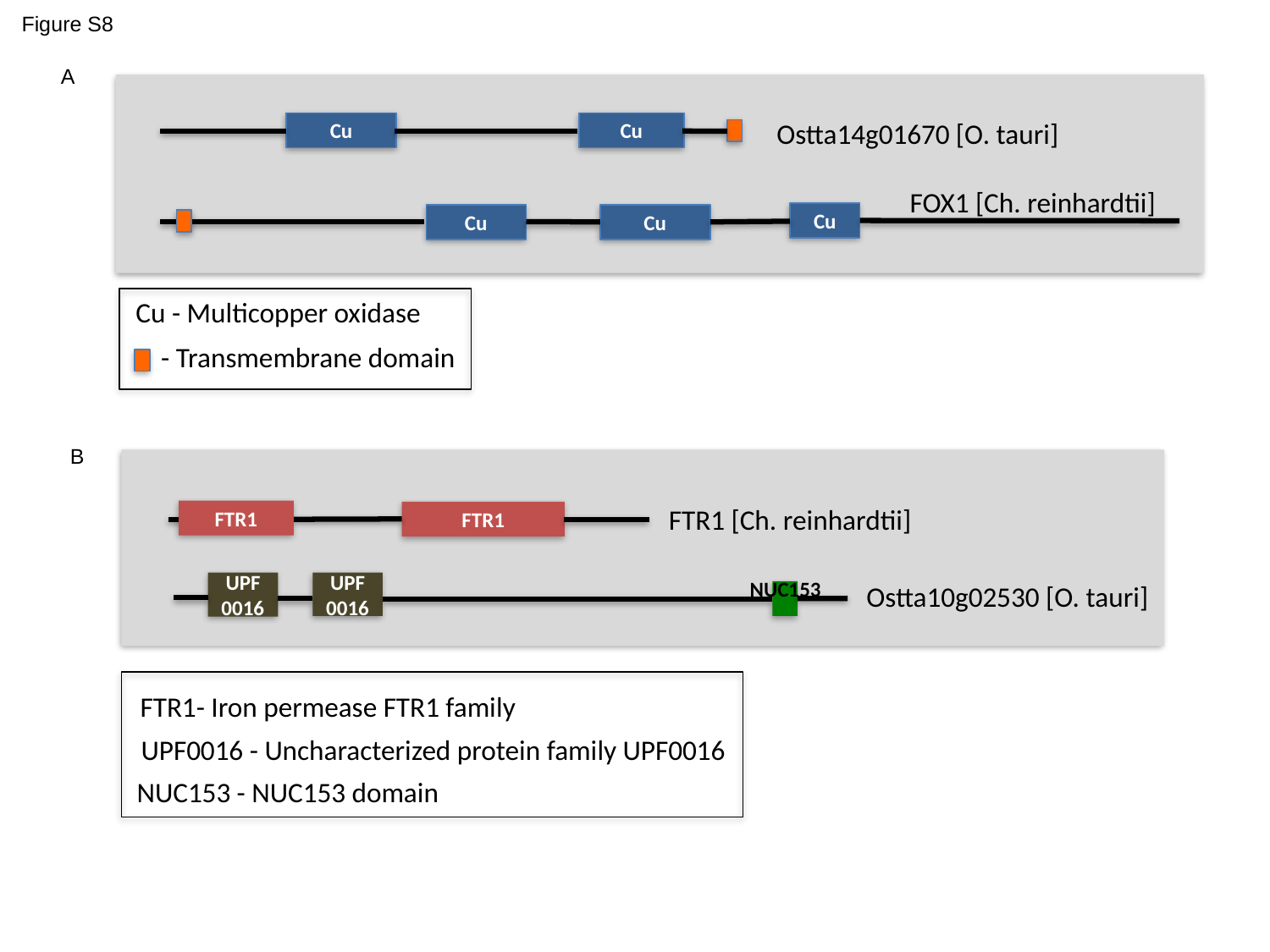

Figure S8
A
Cu
Ostta14g01670 [O. tauri]
Cu
Cu
Cu
FOX1 [Ch. reinhardtii]
Cu
Cu
Cu
Cu
Cu
Cu
Cu - Multicopper oxidase
- Transmembrane domain
B
FTR1 [Ch. reinhardtii]
FTR1
FTR1
NUC153
Ostta10g02530 [O. tauri]
UPF0016
UPF0016
UPF0016
UPF0016
FTR1- Iron permease FTR1 family
UPF0016 - Uncharacterized protein family UPF0016
NUC153 - NUC153 domain
