# Supplementary material for: Ostreococcus tauri is a new model green alga for studying iron metabolism in eukaryotic phytoplankton
Source: BMC Genomics. 2016 May 3;17:319. doi: 10.1186/s12864-016-2666-6 (PMC4855317; doi:10.1186/s12864-016-2666-6)
Supplement: Additional file 12: Figure S10. — The Ot-Fea1 protein contains two Fea1 domains. (A) Multiple alignment of the N- and C-terminal OtFea1 domains and the C. reinhardtii Fea1 protein. R/K-E/D-X-X-E motifs are framed. (B) Domain organization of O. tauri OtFea1, P. tricornutum Isip2a and C. reinhardtii Fea1 as revealed by HMM-HMM comparison, with the Pfam database and the Phobius transmembrane topology and signal peptide predictor. (PPTX 781 kb) [file 12864_2016_2666_MOESM12_ESM.pptx]

## Slide 1
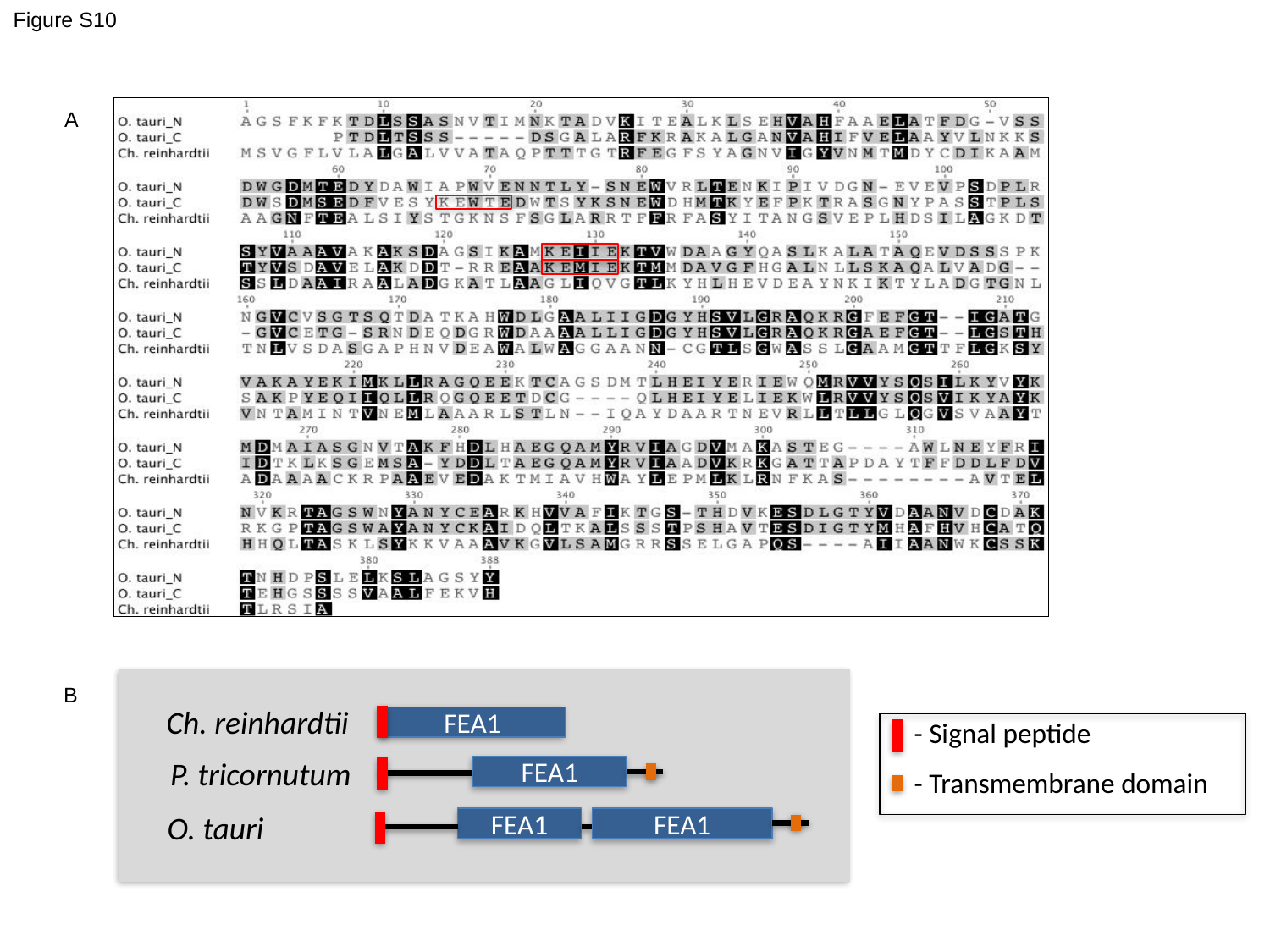

Figure S10
A
B
Ch. reinhardtii
FEA1
- Signal peptide
P. tricornutum
FEA1
- Transmembrane domain
O. tauri
FEA1
FEA1
